# Spatiotemporal dynamics of single cell stiffness in the early developing ascidian chordate embryo

**DOI:** 10.1038/s42003-021-01869-w

**Published:** 2021-03-16

**Authors:** Yuki Fujii, Wataru C. Koizumi, Taichi Imai, Megumi Yokobori, Tomohiro Matsuo, Kotaro Oka, Kohji Hotta, Takaharu Okajima

**Affiliations:** 1 Graduate School of Information Science and Technology, Hokkaido University, Sapporo, Japan; 2 Department of Bioscience and Informatics, Faculty of Science and Technology, Keio University, Yokohama, Japan

**Keywords:** Biophysics, Embryogenesis

## Abstract

During the developmental processes of embryos, cells undergo massive deformation and division that are regulated by mechanical cues. However, little is known about how embryonic cells change their mechanical properties during different cleavage stages. Here, using atomic force microscopy, we investigated the stiffness of cells in ascidian embryos from the fertilised egg to the stage before gastrulation. In both animal and vegetal hemispheres, we observed a Rho kinase (ROCK)-independent cell stiffening that the cell stiffness exhibited a remarkable increase at the timing of cell division where cortical actin filaments were organized. Furthermore, in the vegetal hemisphere, we observed another mechanical behaviour, i.e., a ROCK-associated cell stiffening, which was retained even after cell division or occurred without division and propagated sequentially toward adjacent cells, displaying a characteristic cell-to-cell mechanical variation. The results indicate that the mechanical properties of embryonic cells are regulated at the single cell level in different germ layers.

## Abstract (web summary)

Fujii et al. investigate the stiffness of cells in ascidian embryos from the fertilised egg to the stage before gastrulation. They find two types of cell stiffening, occurring during cell division and in the interphase, the latter of which is associated with the Rho kinase pathway. They conclude that the mechanical properties of early embryonic cells are regulated specifically at the single cell level.

## Introduction

During early embryogenesis, cells undergo dramatic changes in shape, size and polarity, which display a large cell-to-cell (spatial) variation. Recent studies have revealed that, in early embryogenesis characterised by rapid and synchronous cleavage divisions, the developing process is highly regulated by cellular mechanics through the cytoskeleton^1–10^. Furthermore, a close correlation between cell division and force during the subsequent early developmental process has been observed in the zebrafish epiboly^11–13^, epithelium of *Xenopus laevis* embryo^14^, *Drosophila* wing disc^15,16^ and the dorsal midline of the ascidian embryo^17,18^. However, little is known about how individual cells temporarily change their mechanical properties in early embryogenesis after the embryo has divided into two hemispheres such as animal and vegetal poles.

**Fig. 1:**
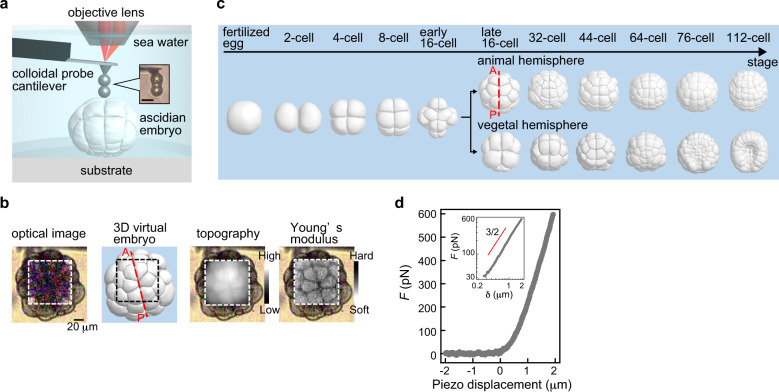
AFM force mapping measurement of the early developing embryo. **a** Schematic of AFM for measurement of the developing ascidian embryo weakly adhered on a dish in seawater. The inset shows an optical microscopic image of the customised colloidal probe cantilever arranging two silica beads from the AFM tip with epoxy glue. Scale bar, 10 μm. **b** Example of a bright-field optical microscopic image and AFM topographic, i.e., relative height *H*, and apparent Young’s modulus *E*, images of the ascidian embryo at around the 32-cell stage. The measured embryonic cells and cell lineage were identified by comparing the AFM images with a 3D model^52^. The values of soft and hard presented here are *ν* = 1.4 and 2.8 of *ν* = log_10_*E* (Pa), respectively. **c** Morphological change in the 3D ascidian embryo model at early developmental stages from the fertilised egg to the 112-cell stage before gastrulation, measured by AFM. Dotted line (red) represents the anterior–posterior line of the embryo. **d** Typical AFM force–distance curve measured in an upper region of a developing embryo sample. As shown in the inset, the force-indentation curve within 2 μm of the indentation depth *δ* followed the power law of 3/2, which corresponds to both conventional and modified Hertzian contact models (‘Methods’).

To address this issue, we investigated the spatiotemporal change in single-cell mechanical properties of the ascidian embryo that exhibits mosaic development^19^ using atomic force microscopy (AFM)^20^ (Fig. 1a) providing a non-destructive measurement to simultaneously determine the topography and stiffness of embryonic cells (‘Methods’). Because the cell lineages of the ascidian embryo have been determined and the embryonic cells are invariant in space during the early developmental stages^21^, the analysed individual cells in embryo samples were identified by comparing AFM topographic and optical microscopic images of samples (‘Methods’ and Fig. 1b). Thus, we obtained information about how individual cells in animal and vegetal hemispheres behave as a mechanical system by mapping the upper surface region of different ascidian embryos.

Here, we show how the stiffness of embryonic cells evolves during early embryogenesis from the fertilised egg to the stage before entering gastrulation (Fig. 1c). The fertilised egg undergoes symmetrical cleavage from the 1-cell to 16-cell stage. Then, the embryo begins to deform anisotropically where the subsequent cell divisions are no longer synchronised in animal and vegetal hemispheres^21^. In the animal hemisphere, the cell division occurs synchronously and a spherical-like shape is still maintained. On the other hand, in the vegetal hemisphere, the shape of the embryo becomes rather flat, and the cells divide asynchronously so that the apical constriction of endodermal cells begins to occur at around the 64-cell stage and initiates gastrulation at the late 112-cell stage^22^. Immunofluorescence revealed that the apical shrinkage in the vegetal hemisphere was relaxed and the apical and circum–apical accumulation of myosin was reduced as the embryo was treated with Rho kinase (ROCK) inhibitor Y-27632^22^. This observation indicated that a Rho pathway controlled the accumulation of myosin in the apical region of the vegetal hemisphere before gastrulation^4,18,22^.

We observed that the stiffness of embryonic cells increased at the onset of cell division and then decreased after the cell divided. Furthermore, we observed in the vegetal hemisphere that cell stiffening occurred even after finishing cell division or without cell division, displaying a characteristic cell-to-cell mechanical variation in the interphase. The Y-27632 treatment experiments indicated that the cell stiffening occurring in the interphase was strongly associated with myosin, which is regulated through the ROCK pathway, while the cell stiffening at the timing of cell division was associated with other pathways.

## Results

### Mechanical properties of embryonic cells measured by AFM

We first checked how well cell mechanical properties in an embryo were estimated by AFM force measurement with a tandemly arranged double-colloidal probe cantilever (Fig. 1a, ‘Methods’). The approaching force–distance curves measured in the upper regions of embryonic cell surfaces were well fitted to the conventional Hertzian contact model^23^ (Fig. 1d, ‘Methods’). The estimated apparent Young’s modulus *E* changed within several hundred nm in the indentation depth, but was almost constant in the further indentation (Supplementary Fig. 1). Thus, we used approaching force–distance curves obtained from the indentation of 1–2 μm to estimate the height *H* and *E* of an embryo, the latter of which was calibrated using the modified Hertzian contact model^24^ considering the tilt angle of the sample surface (‘Methods’). The AFM images acquired at 3 min/frame showed that the cell shapes in *H* and *E* images were identical, and the cell locations were in good agreement with the 3D virtual embryo model determined from confocal microscopic images (Fig. 1b). These results indicated that the *E*-value measured by AFM mainly reflected the mechanical properties of cells that directly contacted the AFM probe. We noticed that the magnitude of *E*-values was largely varied among native embryo samples even under the same condition, which indicated embryo-to-embryo mechanical variation in a chordate sample. The variation was also likely sensitive to the temperature of seawater from which the native chordate samples were collected and the culture day. However, because the origin of the magnitude variation of *E* has not been elucidated, we estimated the average *E* of embryonic cells and the significant difference of *E* between different states or conditions in this study.

### Cells stiffen at the timing of initial symmetrical cell division

**Fig. 2:**
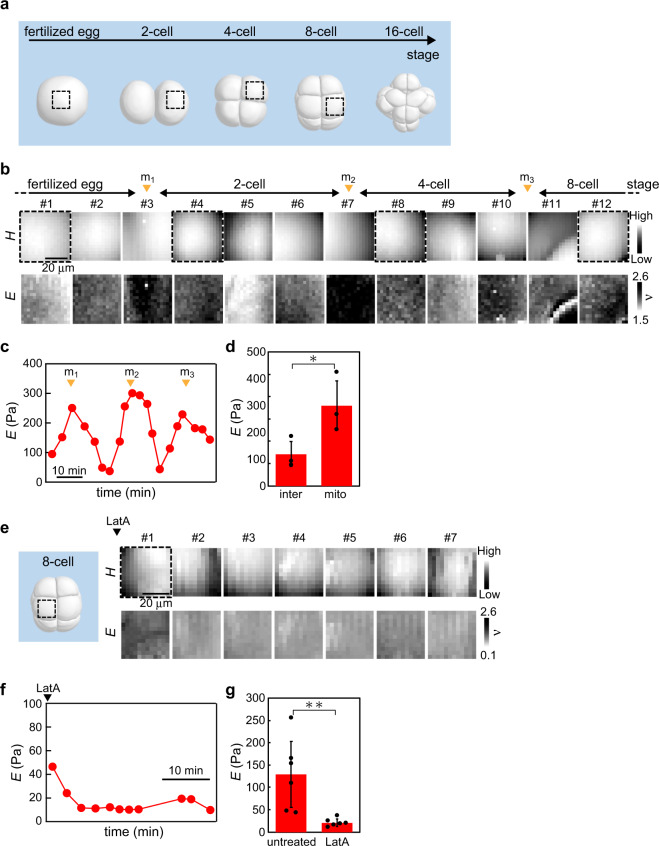
Mapping the ascidian embryo in the initial symmetrical developmental stages. **a** Morphological change in embryonic cells of the 3D model from the fertilised egg (1-cell) to the 16-cell stage. The four dotted squares (black) represent the measured regions that approximately correspond to the four AFM images (#1, #4, #8 and #12) in **b**. **b** Portions of the *H* and *E* images of the embryo mapped at the initial symmetrical developmental stages. The cell division occurred at the times depicted by arrowheads (orange: m_1_, m_2_ and m_3_). **c** Plots of geometrically averaged *E* around the cell centre estimated from all images measured in **b**. **d** *E*-values of embryonic cells at the timing of cell division (mito) and in interphase (inter). Data are geometric mean ± s.d. from *n* = 3 embryos. *P < 0.05. **e** Portions of *H* and *E* images of the embryo mapped at the 8-cell stage after treatment with Latrunculin-A (LatA) as depicted by the arrowhead (black). **f** Plots of geometrically averaged *E* around the cell centre estimated from all images measured in **d**. **g** *E*-values of embryonic cells in untreated and LatA-treated conditions. Data are geometric mean ± s.d. from *n* = 6 embryos. **P < 0.01.

To elucidate the spatiotemporal dynamics of *E* in the ascidian embryo during early cleavage, we first measured *E* of the fertilised egg during the initial symmetric development from the one-cell to the eight-cell stage (Fig. 2a–d). The fertilised egg is known to form cortical actin filaments before the cleavage division^25,26^. The AFM measurement showed that, during the cytokinesis of the first cell division from the one-cell to the two-cell stage (m_1_ in Fig. 2b, c), the cell began to increase *E*, which was more pronounced in the equatorial region when a furrow appeared, and then the blastomeres rapidly reduced *E* after completing cell division (Fig. 2b, #4 and #5). Furthermore, the same increasing behaviour in *E* was observed in subsequent cell divisions from the two-cell to four-cell stage (m_2_ in Fig. 2b, c) and from the four-cell to the eight-cell stage (m_3_ in Fig. 2b, c). The significant change in *E* (Fig. 2d) was reminiscent of the mechanical behaviours observed in single mitotic cells adhered on a substrate in vitro^27,28^ where the cortical actin filaments are formed during mitosis^28,29^.

We also observed that chemical treatment with an actin filament polymerisation inhibitor, Latruncurin A (LatA), led to a significant reduction of *E* that attained a constant value of <50 Pa, which was lower than that in native and unfertilised embryos, and no longer caused cell division (Fig. 2e–g). This result implies an intimate relationship between the change in *E* and the formation of actin filaments.

The average *E* was significantly increased to ~300 Pa at the timing of the cell division (Fig. 2d). The value was the same order of magnitude as the instantaneous elastic modulus of a Xenopus laevis embryo obtained by stress relaxation measurement, which was about tenfold lower than that in vitro single mitotic cells, which is typically around a few kPa^27^. During interphase, the average *E* was minimised to ~100 Pa (Fig. 2c, d), which was comparable with that of the unfertilised egg^24^, indicating that the *E*-value was the lowest stiffness of single cells in the native embryo.

### Animal hemisphere: epidermal cells stiffen at the timing of cell division

**Fig. 3:**
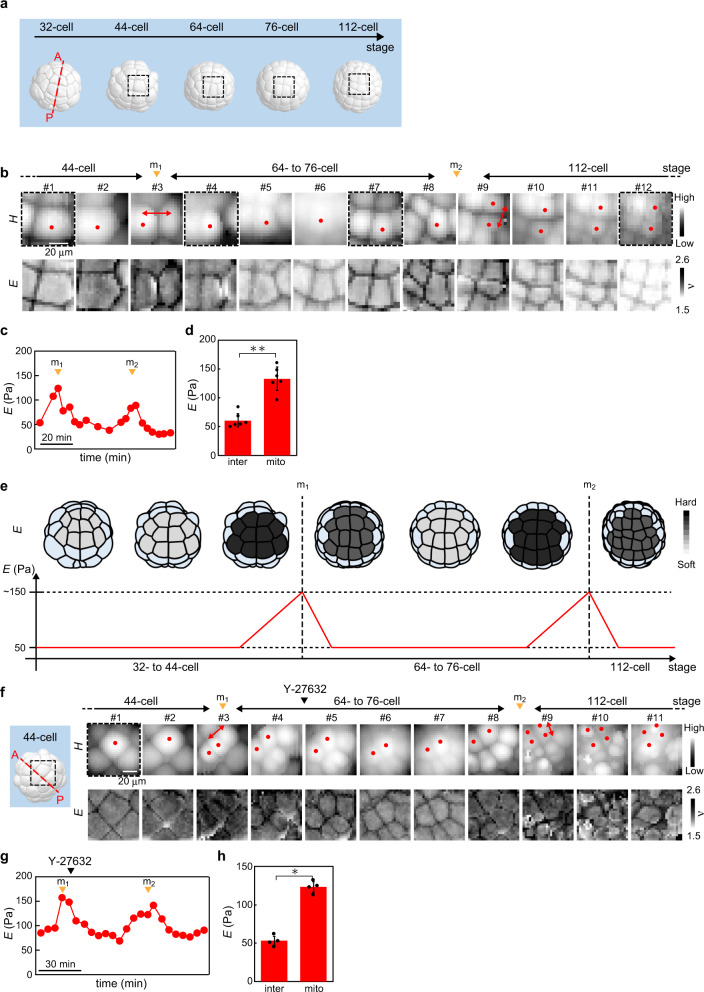
Mapping the ascidian embryo in the animal hemisphere. **a** Morphological change in animal cells of the 3D model from the 32-cell to the 112-cell stage. Dotted line (red) represents the anterior–posterior line of the embryo. Four dotted squares (black) represent measured regions that approximately correspond to the four images (#1, #4, #7 and #12) in **b**. **b** Portions of the *H* and *E* images of the embryo mapped in the animal hemisphere from the 44-cell to 112-cell stage, where cells indicated by dots (red) were tracked. The cell division was observed at times depicted by the arrowheads (orange: m_1_ and m_2_). Double-headed arrows (red) represent the direction of cell division. **c** Plots of geometrically averaged *E* around the centre of cells depicted by the dots (red) in **b**. **d** *E-*values of embryonic cells at the timing of cell division (mito) and in interphase (inter). Data are geometric mean ± s.d. from *n* = 6 embryos. **P < 0.01. **e** Schematic of the change in *E* of animal cells from the 32-cell to the 112-cell stage. The *E*-value of embryonic cells in the 3D model measured by AFM was coloured with a greyscale, showing that the single-cell stiffness increased at the timing of cell division (m_1_ and m_2_) without any apparent cell-to-cell heterogeneities. Cells (light blue) in the 3D model represent those that were not probed by AFM. **f** Portions of the *H* and *E* images of the embryo mapped in the animal hemisphere from the 32-cell to 112-cell stage treated with Y-27632 at 64-cell stage as depicted by the arrowhead (black) where cells depicted as dots (red) were tracked. The dotted square (black) in the 3D embryo model represents the region of the first image (#1). The cell division occurred at times depicted by arrowheads (orange: m_1_ and m_2_). Double-headed arrows represent the direction of cell division. **g** Plots of geometrically averaged *E* around the centre of cells depicted by the dots (red) in **f**. The addition of Y-27632 is depicted by the arrowhead (black). **h** *E*-values of Y-27632-treated cells at the timing of cell division (mito) and in interphase (inter). Data are geometric mean ± s.d. from *n* = 4 embryos. *P < 0.05.

In the later stages from the 32-cell to the 112-cell stage, the significant increase in *E* at the timing of cell division appeared subsequently in the animal hemisphere (m_1_ and m_2_ in Fig. 3a–d). The temporal change in *E* was almost synchronised among epidermal cells at the timing of cell division (Fig. 3b). The time evolution of cell stiffness observed in the animal hemisphere (Fig. 3b–d) is summarised schematically in Fig. 3e.

Approaching cell division, epidermal cells began to increase *E* in the cell–cell boundary region (#1–4 in Supplementary Fig. 2a) and then stimulated furrow formation with a distinct increase of *E*, while the intracellular stiffness was increased gradually (#5 and #6 in Supplementary Fig. 2a). After completing the cell division, *E* decreased rapidly in the intracellular and cell–cell boundary regions (#7–9 in Supplementary Fig. 2a).

**Fig. 4:**
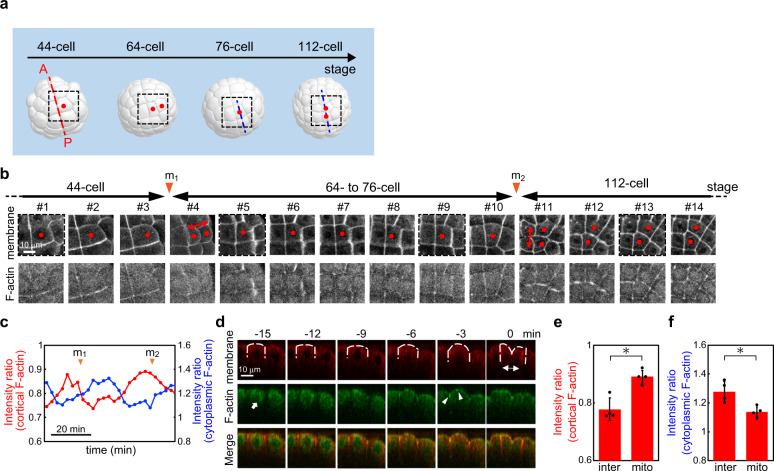
Dynamics of actin filaments (F-actin) in the animal hemisphere cells. **a** Schematic 3D embryo in the animal hemisphere and cell lineage. a6.8 cell divides into a7.15 and a7.16 cells, and a7.16 cell gives rise to a8.31 and a8.32 cells, as depicted by the dots (red). **b** Live imaging of F-actin-binding protein, lifeact-GFP (top) and membrane probe, FM4–64 (bottom) during two rounds of cell division from 44-cells to 112-cells (see also Supplementary Movie 1). Scale bar, 10 μm. Arrowheads show the timing of cell division. The a6.8 cell divided into a7.15 and a7.16 cells, and the a7.16 cell divided into a8.31 and a8.32 cells, as depicted by the dots (red). Two daughter cell pairs were indicated by double-headed arrows (red). Imaged regions were indicated by squares whose colours are the same as those in **a**. **c** Fluorescence intensity ratio of lifeact-GFP both in the plasma membrane (red, corresponding to cortical F-actin) and cytoplasmic regions (blue, corresponding to cytoplasmic F-actin) to FM4–64 in the plasma membrane in the animal pole cells. Arrowheads show the timing of cell division. Data are mean from *n* = 4 cells. **d** Confocal section images of dividing cells (the position of the section was indicated as a blue dotted line in **a**) expressing lifeact-GFP (middle) from 76 cells (see also Supplementary Movie 2) together with cell membrane stained by FM4–64 (top) and merged images (bottom). The timing of cell division is defined as 0 min. The time interval is 3 min. Arrowheads show the accumulation of actin in the apical cell membrane just before cell division. The arrow shows the accumulation of actin in cytoplasmic regions. The cell shape of a7.16 was indicated by white dotted lines. Scale bar, 10 μm. Quantifications of the fluorescence intensity ratio of cortical (**e**) and cytoplasmic (**f**) actin filaments at the timing of cell division (mito) and in the interphase (inter). Data are mean ± s.d. from *n* = 4 cells. *P < 0.05.

Single cells have a round shape during mitosis, which is called mitotic rounding^28,30^. During mitotic rounding, actin filaments accumulate in cortical regions and facilitate cell stiffening^31^. AFM images showed rounding of embryonic cells during mitosis as *E* increased (Supplementary Fig. 2b). Furthermore, the cell stiffening was significantly reduced by treatment with LatA in the animal hemisphere (Supplementary Fig. 3a–c) where the cell stiffening remained unchanged by treatment with DMSO (Supplementary Fig. 3d–f). Furthermore, using a live imaging technique (‘Methods’), we observed clear recruitment and release of actin filaments in cortical regions of the animal hemisphere at the timing of cell division (Fig. 4). The results indicated that the change in *E* observed in epidermis cells at the timing of cell division was associated with the formation and release of cortical actin filaments, the underlying mechanism of which is the same as that in single mitotic cells adhered on the substrate in vitro^28,29^.

We also found that treatment with ROCK inhibitor Y-27632 did not completely suppress the increase in *E* at the timing of cell division (Fig. 3f–h). At the same time, the chemical did not arrest the cell cycle, but the cell shape was different from that in the native embryo (m2 in Fig. 3f), which showed abnormal development as reported previously^32,33^.

### Cell cycle-arrested embryonic cells display a periodic mechanical change

**Fig. 5:**
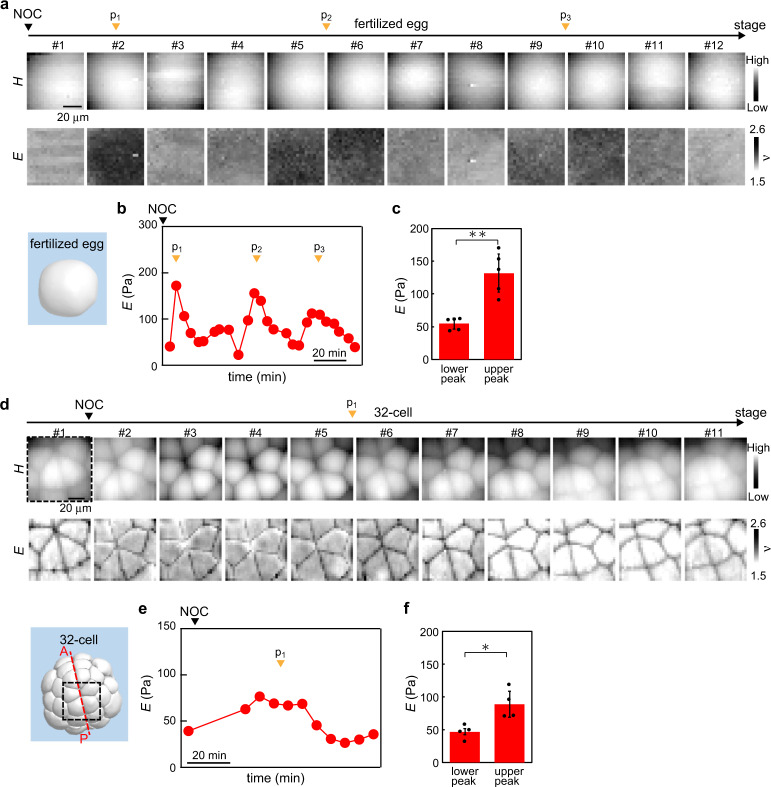
Remarkable change in *E* of embryonic cells occurs even as microtubules depolymerise. **a** Portions of *H* and *E* images of the fertilised egg mapped around the upper region treated with nocodazole (NOC) as depicted by the arrowhead (black). **b** Plots of geometrically averaged *E* estimated from **a**. A remarkable increase in *E* without cell division was observed as depicted by the arrowheads (orange: p_1_, p_2_ and p_3_). The addition of NOC is depicted by the arrowhead (black). **c** *E*-values of NOC-treated cells at the lower and higher peaks. Data are geometric mean ± s.d. from *n* = 5 embryos. **P < 0.01. **d** Portions of *H* and *E* images of the embryo mapped in the animal hemisphere at the 32-cell stage treated with NOC as depicted by the arrowhead (black). Dotted square (black) in the 32-cell stage 3D embryo model represents the measured region in #1 in **c**. **e** Plots of geometrically averaged *E* around the centre of cells estimated from **c**. An increase in *E* was also observed without cell division as depicted by the arrowhead (orange: p_1_). The addition of NOC is depicted by the arrowhead (black). **f** *E*-values of NOC-treated cells at the lower and higher peaks. Data are geometric mean ± s.d. from *n* = 4 embryos. *P < 0.05.

The change in *E* that occurred at the timing of cell division was not necessarily synchronised with the cell division. This was found in an embryo sample treated with nocodazole (NOC) that inhibits the formation of microtubules and facilitates cell cycle arrest. In a cell cycle-arrested fertilised egg, we observed a slight but significant increase and decrease in *E* with a time interval of 30–40 min (Fig. 5a–c), which was still the same order as that in the native sample (Fig. 2b, c). A similar relationship between actin filaments and microtubules has been observed in cells in vitro, so that single cells treated with NOC still accumulated cortical actomyosin and increased rounding force^34^, and the actin filaments exhibited repeated polarisation^35^. Furthermore, we observed such a change in *E* of a NOC-treated embryo with inhibited cell division at later stages, such as the 32-cell stage (Fig. 5d–f). These results suggested that the remodelling of cortical actin filaments in embryonic cells, which contributed to the cell stiffness measured by AFM, was highly conserved during developing processes and less associated with the dynamics of microtubules that facilitate cytokinesis in cell division.

### Vegetal hemisphere: spatiotemporal heterogeneity of single-cell stiffening

**Fig. 6:**
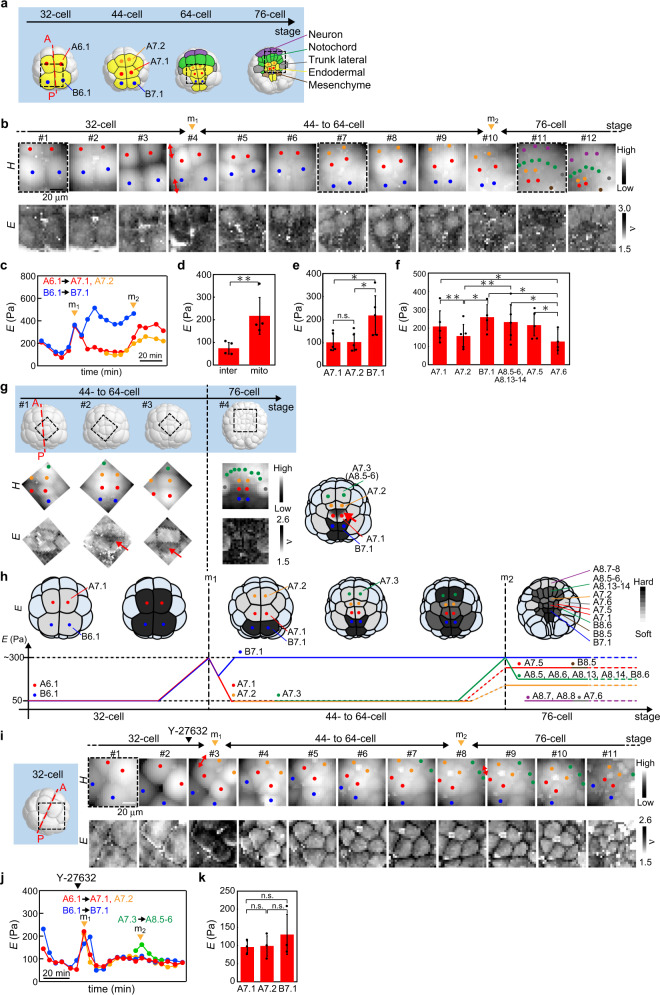
Mapping the ascidian embryo in the vegetal hemisphere. **a** Morphological change of vegetal cells in the 3D model from the 32-cell to 76-cell stage. Dotted line (red) represents the anterior–posterior line of the embryo. Three dotted squares (black) represent the measured regions that approximately correspond to the three images (#1, #7 and #11) in **b**. **b** Portions of the *H* and *E* images of the embryo mapped in the vegetal hemisphere from the 32-cell to 76-cell stage. The cell division occurred at times depicted by arrowheads (orange: m_1_ and m_2_). Double-headed arrows (red) represent the direction of cell division. The colours of dots represent the following cells: red (A7.1), blue (B7.1), orange (A7.2), green (A8.5, A8.6, A8.13 and A8.14) and purple (A8.7 and A8.8). **c** Plots of geometrically averaged *E* around the centre of cells where the colours correspond to those in the dots in **b**. **d** *E*-values of cells at the timing of cell division (mito) from the 32-cell to the 44-cell stage and in the interphase (inter) in the 32-cell stage. Data are geometric mean ± s.d. from *n* = 4 embryos. **P < 0.01. **e** *E*-values of A7.1, A7.2 and B7.1 cells at cell stages from the 44-cell to 64-cell stage. Data are geometric mean ± s.d. from *n* = 5 embryos. *P < 0.05, and n.s. denotes no significant difference. **f** *E*-values of cells observed in the vegetal hemisphere in the 76-cell stage. Data are geometric mean ± s.d. from *n* = 5 embryos. *E*-values of A8.5-6 and A8.13–14 cells were averaged since these small cells were not completely isolated at the single-cell level. *P < 0.05. **g** *H* and *E* images of the embryo mapped in the vegetal hemisphere from the 44-cell to 76-cell stage, displaying subcellular mechanical variations. Dotted squares (black) in the 3D embryo model (upper) represent the measured regions (lower). The colours of dots are the same as those in **b**. In the #1 image, the B7.1 cell (blue) was stiffer than the adjacent A7.1 cells (red). In #2 and #3 images, A7.1 cell (red) began to partly increase stiffness from the region adjacent to the A7.2 cell (orange). In #4, A7.6 cells (grey) were softer than the others, and A7.2 cells were still softer than A7.1 cells and A8.5, A8,6, A8.13 and A8.14 cells (green). **h** Schematic of the change of *E* in vegetal cells from the 32-cell to 76-cell stage. The *E*-value of embryonic cells in the 3D model measured by AFM was coloured with grayscale, showing cell-to-cell mechanical heterogeneity. Cells (light blue) in the 3D model represent those that were not probed by AFM. The colours of dots are the same as those in the dots in **b**. **i** Portions of *H* and *E* images of the embryo mapped in the vegetal hemisphere from the 32-cell to 76-cell stage treated with Y-27632 as depicted by the arrowhead (black). Dotted square (black) in the 3D embryo model represents the region measured in #1. The cell division occurred at times depicted by arrowheads (orange: m_1_ and m_2_). Double-headed arrows represent the direction of cell division. The colours of dots are the same as those in the dots in **b**. **j** Plots of geometrically averaged *E* around the centre of cells depicted by the dots in **i**. The addition of Y-27632 is depicted by the arrowhead (black). **k** *E*-values of Y-27632-treated A7.1, A7.2 and B7.1 cells at cell stages from the 44-cell to 64-cell stage. Data are geometric mean ± s.d. from *n* = 3 embryos. n.s. denotes no significant difference.

We observed complex behaviour of *E* in the vegetal hemisphere (Fig. 6a–h and Supplementary Fig. 4) rather than in the animal hemisphere, showing that the mechanical properties of embryonic cells were independently regulated in different germ layers. First, at cell stages from the 32-cell to the 44-cell stage (#1–6 in Fig. 6b), we tracked a set of adjacent cells, such as A6.1 and B.6.1 cells, which divide into A7.1 and A7.2 cells, and B7.1 and B7.2 cells, respectively. The observation showed that both A6.1 and B6.1 cells began to increase *E* while approaching cell division (m_1_ in Fig. 6b–d), which was similar to the mechanical behaviours observed in the animal hemisphere (Fig. 3b–d). Importantly, after completing the cell division, the evolution of *E* was significantly different between A7.1 and B7.1 cells: in the A7.1 cell, the increased *E* was rapidly reduced, while in the B7.1 cell, the increased *E* was maintained until the later cell stages towards gastrulation (Fig. 6c, e).

Next, we tracked a set of adjacent cells, such as A7.3 (#5–12 in Supplementary Fig. 4), at the later cell stages from the 64-cell to the 76-cell stage in addition to A7.1, A7.2 and B7.1 cells (#7–12 in Fig. 6b). In the 64-cell stage, from which cell fate is determined (Fig. 6a)^36,37^, A7.3 cells adjacent to A7.2 cells began to increase *E* approaching cell division into A8.5 and A8.6 (pre-notochord) cells (Supplementary Fig. 4b–d), which was similar behaviour to B7.1 cells (Fig. 6f). At the same time of the stiffening of A7.3 cells, both A7.1 and A7.2 cells began to increase *E* (Supplementary Fig. 4b, c). Interestingly, the stiffening of A7.1 and A7.2 cells occurred in the subcellular region and not with accompanying cell division (#2 and #3 arrows in Fig. 6g), and the rate of cell stiffening was different between them (Fig. 6c, f and Supplementary Fig. 4c). Apparently, *E* was enhanced sequentially in the direction from posterior (B7.1 cells) to anterior (A7.1. and A7.2 cells) in the vegetal hemisphere (Fig. 6b, c and Supplementary Fig. 4).

When entering the 76-cell stage, the A7.2 cell was softer than the surrounding cells such as A7.1, A8.5-6 and A8.13–14 cells (#4 in Fig. 6g, f), although these cells attained stiffness higher than ~100 Pa in the interphase of the 44-cell stage (Fig. 6f). In addition, as shown in Fig. 6f, we found that A7.6 cells (trunk lateral cells) retained an almost unchanged *E* (#11–12 in Fig. 6b), which were significantly softer than pre-notochord cells such as A8.5-6 and A8.13–14 cells (#12 in Fig. 6b) and pre-endoderm cells such as A7.5 (#11 and #12 in Fig. 6b and #4 in Figs. 6g) and B7.1 (#5 and #6 in Fig. 6b) cells. It was also observed that trunk lateral cells were softer than pre-mesenchyme such as B8.5 cells (#11 and #12 in Fig. 6b and #4 in Fig. 6g). Furthermore, A8.7-8 cells (pre-neuron cells) (#11 and #12 in Fig. 6b and #4 in Fig. 6g) were observed to retain an unchanged *E* that was similar to trunk lateral cells. These results indicated that the change in *E* was different among cells with different cell fates before cell differentiation. The time evolution of cell stiffness observed in the vegetal hemisphere (Fig. 6b–g) is summarised schematically in Fig. 6h.

We found that *E* maintained at a higher value in B7.1 cell was significantly suppressed by treatment with Y-27632 (Fig. 6i–k), which was different from the behaviours observed in the change of *E* at the timing of cell division (Fig. 3f–h). In addition, the Y-27632 treatment suppressed stiffening of A7.1 and A7.2 cells occurring without cell division at the 76-cell stage (Fig. 6i–k). These results indicated that the cell stiffening occurring without cell division in the interphase was strongly associated with myosin, which is regulated through the ROCK pathway, while the cell stiffening at the timing of cell division was associated with other pathways.

## Discussion

During mitosis in fertilised eggs, actin filaments accumulate in the cortical region and contraction ring^25,26^. Our AFM analyses showed that the fertilised egg exhibited a remarkable increase in *E*, which was more pronounced in the equatorial region when a furrow appeared (#1–5 in Fig. 2b) similarly to that during the division of cells adhered on a substrate in vitro^27^. Similar cell stiffening at the timings of cell division was observed in subsequent stages from the two-cell to eight-cell stage (#6–12 in Fig. 2b) and the stages until the 112-cell stage before entering gastrulation in both animal (Fig. 3b, c) and vegetal (Fig. 4b, c) hemispheres. Furthermore, we observed that depolymerisation of actin filaments led to a significant reduction in *E* (Fig. 2e–g and Supplementary Fig. 3a–c) and that clear recruitment and release of actin filaments in cortical regions of the animal hemisphere at the timing of cell division (Fig. 4). Based on the results, we concluded that AFM force mapping measurement can mechanically monitor the remodelling of cortical actin filaments in embryonic cells during early embryogenesis.

The total stress operating in the developing embryo includes ‘active’ stress defined as contractile traction force or tension exerted in cells and between adjacent cells and ‘passive’ stress as the intracellular stiffness^38^. Previous studies have revealed that hydrodynamic pressure and tension due to the actomyosin cortex lead to the mitotic cell rounding of single cells^28,39^ and a confined cell system^34^. In stages that embryonic cells were tightly confined as adjacent cells, we observed that the mitotic cell rounding appeared as *E* increased (Supplementary Fig. 2). Therefore, it is reasonable to consider that the indentation force measured by AFM involves tensile force and hydrodynamic pressure in addition to force resulting from the elastic modulus of cells. Assuming that *T* is measured predominantly by AFM at a shallow indentation depth with an indentation force of less than 200 pN, we found that *T* was correlated positively with *E* (Supplementary Fig. 5). This correlation between *E* and *T* does not contradict our consideration that the observed change in *E* of mitotic cells involves the change in the tensile forces and hydrodynamic pressure reported in the previous studies^28,34,39,40^. Therefore, *E* estimated in this study is regarded as a measure of total stress operating in the developing embryo.

**Fig. 7:**
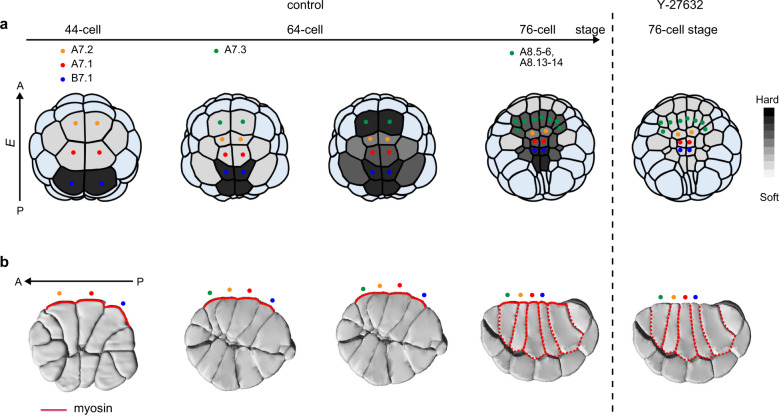
Schematic of the relationship between actomyosin structures and single-cell stiffening in the ascidian vegetal hemisphere from the 44-cell to 76-cell stage. **a** Magnitude of *E* represented by a greyscale (upper). Cell stiffening was reduced completely by Y-27632 treatment. **b** Myosin began to accumulate in apical regions from the 44-cell stage and was then recruited to both circum–apical and basolateral regions from the 64-cell to 76-cell stage, as reported in Ref^4^.. Solid curves (red) represent myosin accumulated in apical regions, while the dotted curves (red) represent myosin accumulating in the basolateral region. Dotted colours represent those in **a**. After treatment with Y-27632, myosin in apical regions was disrupted, but not in the basolateral region.

The dynamic change of *E* in embryonic cells during early development from the fertilised egg to the 112-cell stage before entering gastrulation can be classified into two types. The first is the ROCK-independent cell stiffening that *E* of cells increases at the onset of cell division and then decreases after finishing the cell division (Figs. 2b–d,  3b–d, and  6b–d). This was commonly observed in both animal and vegetal hemispheres as well as in symmetrical developing stages. The second type is the ROCK-associated cell stiffening, which was observed only in the vegetal hemisphere (Figs. 6b–h and  7): The increased *E* of cells did not decrease even after finishing the cell division and was consequently maintained in the interphase as shown in the division of B6.1 (Fig. 6b, c) and A7.3 cells (Supplementary Fig. 4b, c), while *E* of cells increased without cell division and was consequently maintained in the interphase as shown in A7.1 and A7.2 cells (Fig. 6b, c).

In this context, previous studies have shown that Y-27632 treatment strongly inhibits apical and circum–apical myosin, but not basolateral myosin where myosin that facilitates the apical construction^22^ began to accumulate in apical regions from around the 64-cell stage and then in the basolateral region (Fig. 7). These results suggest that the change of *E* in the second type observed in the vegetal hemisphere reflects the apical formation of actomyosin, which is highly regulated in subcellular regions in the direction from posterior (B7.1 cell) to anterior (A7.1. and A7.2 cells) (Fig. 6b, c, g, h and Supplementary Fig. 4).

We noticed that no apparent cell-to-cell variation in myosin density in apical regions has been observed in previous studies by immunofluorescence^22^, implying that the apical actomyosin network is remodelled without changing the myosin density and facilitates a mechanically stabilised structure. Thus, we consider that AFM provides a complementary approach to quantitative investigations of dynamic structural changes in embryonic cells during the developmental process.

Previous studies have reported that the number distribution of cell mechanical properties exhibits a large variation in single cells^41,42^ and the cell stiffness and tension are very broadly varied over a long intercellular distance in a cell monolayer^43–46^. In contrast, the embryonic cells in the vegetal hemisphere exhibited broad spatiotemporal heterogeneity occurring at the single-cell level. From the 64-cell to 76-cell stage, at the timing that A7.2 cells (endodermal cells) began to increase *E*, the adjacent cells such as A8.5-6 and A8.13–14 cells (pre-notochord cells) also increased *E* (Fig. 6a–h). It is known that, at the 64-cell stage, notochord precursor cells begin to express a specific gene, so-called brachyury, which is a key transcription factor for notochord differentiation in ascidians^47^. Our results implied that the mechanical properties of embryonic cells may be associated with gene expression, as reported in single cells^48^.

### Conclusions

Our AFM measurements of the ascidian embryo showed single-cell stiffening and cell rounding at the timing of cell division, which have been commonly observed in single mitotic cells in vitro and monolayer cultures on a substrate. Furthermore, at stages from the 32-cell to the 112-cell stage before entering gastrulation, we found characteristic cell stiffening and softening in the vegetal hemisphere, which were different from those in the animal hemisphere. Treatment with Y-27632 showed that the single-cell stiffening without cell division observed only in the vegetal hemisphere was associated with myosin that is regulated through the ROCK pathway, while the single-cell stiffening at the timing of cell division in both hemispheres was mediated via other pathways. Taken together with the previous immunofluorescence observations indicating that apical and circum–apical myosin were specifically inhibited by Y-27632, but no apparent spatial variation in myosin density was observed, we concluded that the spatiotemporal change in *E* of the vegetal hemisphere reflected the formation and remodelling of the apical actomyosin network. The direct AFM measurements of cell stiffness in the ascidian embryo, a model of early chordate development, can facilitate a better understanding of how embryonic single cells in animals are spatiotemporally regulated via mechanical cues.

## Methods

### Ascidian samples

Adult *Ciona intestinalis* type A (*Ciona robusta*) was collected in the vicinity of the Maizuru Fisheries Research Station (Kyoto University) and Misaki Marine Biological Station (The University of Tokyo) through the National BioResource Project (NBRP). Eggs and sperm were obtained by dissection of the ascidian gonoducts. Unfertilised eggs were dechorionated by natural seawater (Nihon Aquarium) containing 1.0% sodium thioglycolate (Wako Pure Chemical Industries Ltd.) and 0.05% actinase E (Rikaken Pharmaceutical Co. Ltd.). After washing the dechorionated eggs in seawater several times, they were artificially inseminated and reared on 0.1% gelatin (Wako Pure Chemical Industries Ltd.)-coated dishes in seawater at 18 °C for 3 hpf (hour post-fertilised). For AFM measurements, the embryos were weakly attached to a cell culture dish (Iwaki) in seawater to prevent unexpected translational movement during the experiments. During the preparation, the embryos occasionally failed to undergo normal development during the early developmental stage. Therefore, we showed experimental data of embryos developed until the gastrula stage.

### AFM measurements

A customised atomic force microscope^46^ was used to map the relative height and apparent Young’s modulus *E* of ascidian embryo samples. The atomic force microscope was mounted on an upright optical microscope (Eclipse FN1, Nikon) with a liquid-immersion objective lens (plan fluor, ×10, Nikon) for the optical lever system, as reported previously^46^. We used a rectangular cantilever (Biolever-mini, BL-AC40TS-C2, Olympus) with a nominal spring constant of <0.1 N/m. Before the experiments, the spring constant of the cantilever was determined in liquid environments using a thermal fluctuation procedure built in a commercial atomic force microscope (MFP-3D, Asylum Research). To achieve a well-defined contact geometry and prevent the contact between the cantilever beam and the sample surface, two silica beads with a radius *R* of ca. 5 μm (Funakoshi) were arranged tandemly from the AFM tip with epoxy glue (Nichiban).

The AFM force mapping measurements were conducted in a part of the upper region of the embryo at 18 °C with a piezoscanner (E-761, Physik Instruments) controlled with LabVIEW software (National Instruments), where the embryo samples were weakly adhered on a dish so that abnormal cell division was highly prevented. However, the weak adhesion occasionally caused small unexpected fluctuations. Thus, the mapped positions by AFM were changed manually to trace the approximately same region of the embryo in the developing process according to the sample fluctuation. The scan range was about 72 × 72 μm with a spacing of 3 μm. The acquisition time for a single mapping image was ~3 min with a slow scan speed, which reduced the unexpected movement of embryo samples. The maximum loading force was set at 0.6 nN. The cells mapped by AFM were identified using the Four-Dimensional Ascidian Body Atlas database^21^.

### Estimate of the apparent Young’s modulus and tension

The *E* of embryonic cells was estimated from approaching force–distance curves using the modified Hertz contact model^24^. According to the conventional Hertz contact model^23^, as a spherical probe with a radius of *R* indents perpendicular to the flat surface of a cell with *E* with an indentation depth of *δ*, the loading force *F* is given by

1 $$F = \frac{{4R^{1/2}E}}{{3\left( {1 - \nu _{\mathrm{p}}^2} \right)}}\delta ^{3/2},$$

where *ν*_p_ is the Poisson’s ratio of the cell, which was assumed to be 0.5. Because the surface of the embryo samples was not flat but tilted, *F* changed depending on the tilt angle *θ* of the sample surface that can be estimated from an AFM topographic image. In this case, *E* for soft materials with <~10 kPa was calibrated with a modified Hertz contact model ^24^:

2 $${\it{F}} = \frac{{4R^{1/2}\left( {E\cos ^{5/2}\theta } \right)}}{{3\left( {1 - \nu _{\mathrm{p}}^2} \right)}}\delta ^{3/2}.$$

Because the modified Hertz contact model was valid for *θ* <40°^24^, we discussed the phenomena observed in mapping regions that satisfied *θ* <40°. Images of *E* were scaled with *ν* = log_10_*E* (Pa).

According to the liquid droplet model^49,50^, the cell–cortex tension *T* was estimated by the following equation:

3 $$F = 2T\left( {\frac{1}{R} + \frac{1}{r}} \right)\left( {2\pi R} \right)\delta$$

where *r* is the cell radius, which was assumed to be 15 μm. To estimate the apparent *T* of embryonic cells, we fitted with Eq. (3) approaching force–distance curves of <200 pN, typically corresponding to an indentation of less than 1.0 μm, which was one order of magnitude smaller than the cell diameter.

### Chemical treatments

To inhibit actin filament polymerisation, the embryos were treated with 0.1–0.2 μM latrunculin-A (LatA, Sigma-Aldrich) during time-lapse AFM mapping measurements. To inhibit Rho kinase (ROCK) and thereby suppress myosin II phosphorylation, the embryos were treated with 100 μM Y-27632 (Sigma-Aldrich) during time-lapse AFM mapping measurements. To inhibit the formation of microtubules and thereby halt cell division, the embryos were treated with 5 μM nocodazole (NOC, Sigma-Aldrich) during time-lapse AFM mapping measurements. To quantify the chemical dependence of *E*, we analysed the treated cells after incubation for at least 10 min with latA and 20 min with Y-27632.

### Live imaging of cytoskeletons

For cytoskeleton imaging, lifeact-GFP was gifted by Dr. Benoit Godard of IST Austria. Lifeact-GFP is an actin marker for the visualisation of actin filaments (F-actin)^51^. The mRNA was synthesised by using mMESSAGE mMACHINE Kit (Ambion). According to the protocol provided by the company, reaction mixture (2× NTP/CAP 10 µL, 10× reaction buffer 2 µL, enzyme mix 2 µL, linearised DNA 1 µg and nuclease-free water to 20 µL) was prepared in 1.5-mL tube. The reaction mixture was incubated at 37 °C for 2 h. TURBO DNase was added and incubated at 37 °C for 15 min. Nuclease-free water and LiCl precipitation solution were added and chilled for 30 min at –20 °C. The reaction mixture was centrifuged at 4 °C for 15 min at maximum speed to pellet the RNA. The pellet was washed twice in 70% ethanol and centrifuged at maximum speed for 5 min. After removing 70% ethanol, nuclease-free water was added and stored at –20 °C.

The mRNA-encoding lifeact-GFP was injected into unfertilised eggs. After fertilisation, embryos were incubated for 3 h at 20 °C and mounted on a glass-based dish (IWAKI). The embryos were immersed into seawater with FM4–64 to stain cell membrane. Time-lapse 3D imaging was performed using a confocal laser scanning microscope (fv1000, Olympus) equipped with a ×40 oil-immersion lens. During the observation, the temperature was kept using a temperature controller. Step size of the *z* axis was set at 2 µm, and 20–30 image confocal stacks were acquired per sample. The time interval was 3 min. These images were reconstructed to 3D image by software (Imaris, bitplane).

### Fluorescence image analysis

Fluorescence images of F-actin (lifeact-GFP) and cell membrane (FM4–64) were loaded and converted to greyscale images. Cell membrane region was manually determined with a threshold using membrane fluorescence images according to the Otsu method. Then, the intensity ratios of F-actin (lifeact-GFP) in the cell membrane region (corresponding to cortical F-actin) and in the cytoplasmic region (corresponding to cytoplasmic F-actin) to the cell membrane (FM4–64) were calculated during the developmental process.

### Statistics and reproducibility

For data analysis, an unpaired two-tailed Student’s *t* test was used to determine *P* values. The *P* values <0.05 were considered to be significant. The number of individual experiments is in the corresponding figure legend.

### Reporting summary

Further information on research design is available in the Nature Research Reporting Summary linked to this article.

## Supplementary information

Peer Review File

Supplementary Information

Description of Additional Supplementary Files

Supplementary Movie 1

Supplementary Movie 2

Supplementary Data 1

Reporting Summary

## Data Availability

All the data for generating the graphs in all the main figures are included in Supplementary Data 1. The image sets in the main figures and any remaining information are available from the corresponding author on reasonable request.

## Code availability

Igor Pro (version 8, WaveMetrics) and Image J (version 1.52, NIH) were used for the AFM data analysis. The detailed protocols and codes are available from the corresponding author on reasonable request.

## References

[1] Martin P, Parkhurst SM (2004). Parallels between tissue repair and embryo morphogenesis. Development.

[2] Fernandez-Gonzalez R, Simoes Sde M, Roper JC, Eaton S, Zallen JA (2009). Myosin II dynamics are regulated by tension in intercalating cells. Dev. Cell.

[3] Mammoto T, Ingber DE (2010). Mechanical control of tissue and organ development. Development.

[4] Lecuit T, Lenne PF, Munro E (2011). Force generation, transmission, and integration during cell and tissue morphogenesis. Annu Rev. Cell Dev. Bi.

[5] Levayer R, Lecuit T (2012). Biomechanical regulation of contractility: spatial control and dynamics. Trends Cell Biol..

[6] Miller CJ, Davidson LA (2013). The interplay between cell signalling and mechanics in developmental processes. Nat. Rev. Genet..

[7] Takeichi M (2014). Dynamic contacts: rearranging adherens junctions to drive epithelial remodelling. Nat. Rev. Mol. Cell Biol..

[8] Petridou NI, Spiro Z, Heisenberg CP (2017). Multiscale force sensing in development. Nat. Cell Biol..

[9] Roca-Cusachs P, Conte V, Trepat X (2017). Quantifying forces in cell biology. Nat. Cell Biol..

[10] Miroshnikova YA (2018). Adhesion forces and cortical tension couple cell proliferation and differentiation to drive epidermal stratification. Nat. Cell Biol..

[11] Campinho P (2013). Tension-oriented cell divisions limit anisotropic tissue tension in epithelial spreading during zebrafish epiboly. Nat. Cell Biol..

[12] Brunet T (2013). Evolutionary conservation of early mesoderm specification by mechanotransduction in Bilateria. Nat. Commun..

[13] Xiong FZ (2014). Interplay of cell shape and division orientation promotes robust morphogenesis of developing epithelia. Cell.

[14] Woolner S, Papalopulu N (2012). Spindle position in symmetric cell divisions during epiboly is controlled by opposing and dynamic apicobasal forces. Dev. Cell.

[15] LeGoff L, Rouault H, Lecuit T (2013). A global pattern of mechanical stress polarizes cell divisions and cell shape in the growing Drosophila wing disc. Development.

[16] Mao YL (2013). Differential proliferation rates generate patterns of mechanical tension that orient tissue growth. EMBO J..

[17] Negishi T, Yasuo H (2015). Distinct modes of mitotic spindle orientation align cells in the dorsal midline of ascidian embryos. Dev. Biol..

[18] Hashimoto H, Munro E (2018). Dynamic interplay of cell fate, polarity and force generation in ascidian embryos. Curr. Opin. Genet. Dev..

[19] Nishida H (2002). Patterning the marginal zone of early ascidian embryos: localized maternal mRNA and inductive interactions. Bioessays.

[20] Krieg M (2019). Atomic force microscopy-based mechanobiology. Nat. Rev. Phys..

[21] Hotta K (2007). A web-based interactive developmental table for the ascidian Ciona intestinalis, including 3D real-image embryo reconstructions: I. from fertilized egg to hatching larva. Dev. Dyn..

[22] Sherrard K, Robin F, Lemaire P, Munro E (2010). Sequential activation of apical and basolateral contractility drives ascidian endoderm invagination. Curr. Biol..

[23] Landau, L. D. & Lifshits, E. M. *Theory of Elasticity*, third edn. (Pergamon Press, Oxford Oxfordshire, 1986).

[24] Fujii, Y. & Okajima, T. Calibrating the Young’s modulus of soft materials with surface tilt angle measured by atomic force microscopy. *AIP Adv*. **9**, 015028 (2019).

[25] Wong GK, Allen PG, Begg DA (1997). Dynamics of filamentous actin organization in the sea urchin egg cortex during early cleavage divisions: implications for the mechanism of cytokinesis. Cell Motil. Cytoskeleton.

[26] Reymann, A. C., Staniscia, F., Erzberger, A., Salbreux, G. & Grill, S. W. Cortical flow aligns actin filaments to form a furrow. *eLife* **5**, e17807 (2016). 10.7554/eLife.17807 PMC5117871 27719759

[27] Matzke R, Jacobson K, Radmacher M (2001). Direct, high-resolution measurement of furrow stiffening during division of adherent cells. Nat. Cell Biol..

[28] Stewart MP (2011). Hydrostatic pressure and the actomyosin cortex drive mitotic cell rounding. Nature.

[29] Salbreux G, Charras G, Paluch E (2012). Actin cortex mechanics and cellular morphogenesis. Trends Cell Biol..

[30] Ramkumar N, Baum B (2016). Coupling changes in cell shape to chromosome segregation. Nat. Rev. Mol. Cell Biol..

[31] Stewart MP, Toyoda Y, Hyman AA, Muller DJ (2011). Force probing cell shape changes to molecular resolution. Trends Biochem. Sci..

[32] Pacquelet A, Uhart P, Tassan JP, Michaux G (2015). PAR-4 and anillin regulate myosin to coordinate spindle and furrow position during asymmetric division. J. Cell Biol..

[33] Chanet S, Sharan R, Khan Z, Martin AC (2017). Myosin 2-induced mitotic rounding enables columnar epithelial cells to interpret cortical spindle positioning cues. Curr. Biol..

[34] Sorce B (2015). Mitotic cells contract actomyosin cortex and generate pressure to round against or escape epithelial confinement. Nat. Commun..

[35] Fink J (2011). External forces control mitotic spindle positioning. Nat. Cell Biol..

[36] Nishida H (1997). Cell fate specification by localized cytoplasmic determinants and cell interactions in ascidian embryos. Int. Rev. Cytol..

[37] Kumano G, Nishida H (2007). Ascidian embryonic development: an emerging model system for the study of cell fate specification in chordates. Dev. Dyn..

[38] Gross P, Kumar KV, Grill SW (2017). How active mechanics and regulatory biochemistry combine to form patterns in development. Annu. Rev. Biophys..

[39] Fischer-Friedrich E, Hyman AA, Julicher F, Muller DJ, Helenius J (2014). Quantification of surface tension and internal pressure generated by single mitotic cells. Sci. Rep..

[40] Smeets B, Cuvelier M, Pesek J, Ramon H (2019). The effect of cortical elasticity and active tension on cell adhesion mechanics. Biophys. J..

[41] Cai P (2013). Quantifying cell-to-cell variation in Power-Law rheology. Biophys. J..

[42] Cai P (2017). Temporal variation in single-cell Power-Law rheology spans the ensemble variation of cell population. Biophys. J..

[43] Tambe DT (2011). Collective cell guidance by cooperative intercellular forces. Nat. Mater..

[44] Garcia S (2015). Physics of active jamming during collective cellular motion in a monolayer. Proc. Natl Acad. Sci. USA.

[45] Park JA (2015). Unjamming and cell shape in the asthmatic airway epithelium. Nat. Mater..

[46] Fujii Y (2019). Spontaneous spatial correlation of elastic modulus in jammed epithelial monolayers observed by AFM. Biophys. J..

[47] Nishida H (2005). Specification of embryonic axis and mosaic development in ascidians. Dev. Dyn..

[48] Toyoda, Y. et al. Genome-scale single-cell mechanical phenotyping reveals disease-related genes involved in mitotic rounding. *Nat. Commun*. **8**, 1–11 (2017). 10.1038/s41467-017-01147-6 PMC5668354 29097687

[49] Lomakina EB, Spillmann CM, King MR, Waugh RE (2004). Rheological analysis and measurement of neutrophil indentation. Biophys. J..

[50] Krieg M (2008). Tensile forces govern germ-layer organization in zebrafish. Nat. Cell Biol..

[51] Riedl J (2008). Lifeact: a versatile marker to visualize F-actin. Nat. Methods.

[52] Tassy O (2010). The ANISEED database: digital representation, formalization, and elucidation of a chordate developmental program. Genome Res..

