# Supplementary material for: Spatiotemporal dynamics of single cell stiffness in the early developing ascidian chordate embryo
Source: Commun Biol. 2021 Mar 16;4:341. doi: 10.1038/s42003-021-01869-w (PMC7966737; doi:10.1038/s42003-021-01869-w)
Supplement: Supplementary file 2 — Supplementary Information [file 42003_2021_1869_MOESM2_ESM.pdf]

Supplementary Information

Spatiotemporal dynamics of single cell stiffness in the early developing ascidian chordate embryo

Yuki Fujii, Wataru C. Koizumi, Taichi Imai, Megumi Yokobori, Tomohiro Matsuo, Kotaro Oka, Kohji Hotta, and Takaharu Okajima

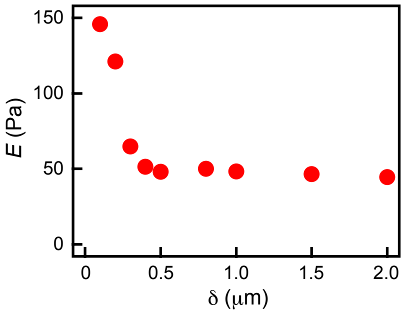

Supplementary Figure 1 The apparent Young's modulus E of an unfertilised egg estimated from the force-indentation curve within the indentation depth δ with Eq. 2. The measurement was examined around the upper region of the egg. The estimated E was largely varied within 0.5 μm in δ and almost constant to be ca. 50 Pa for δ larger than 0.5 μm.

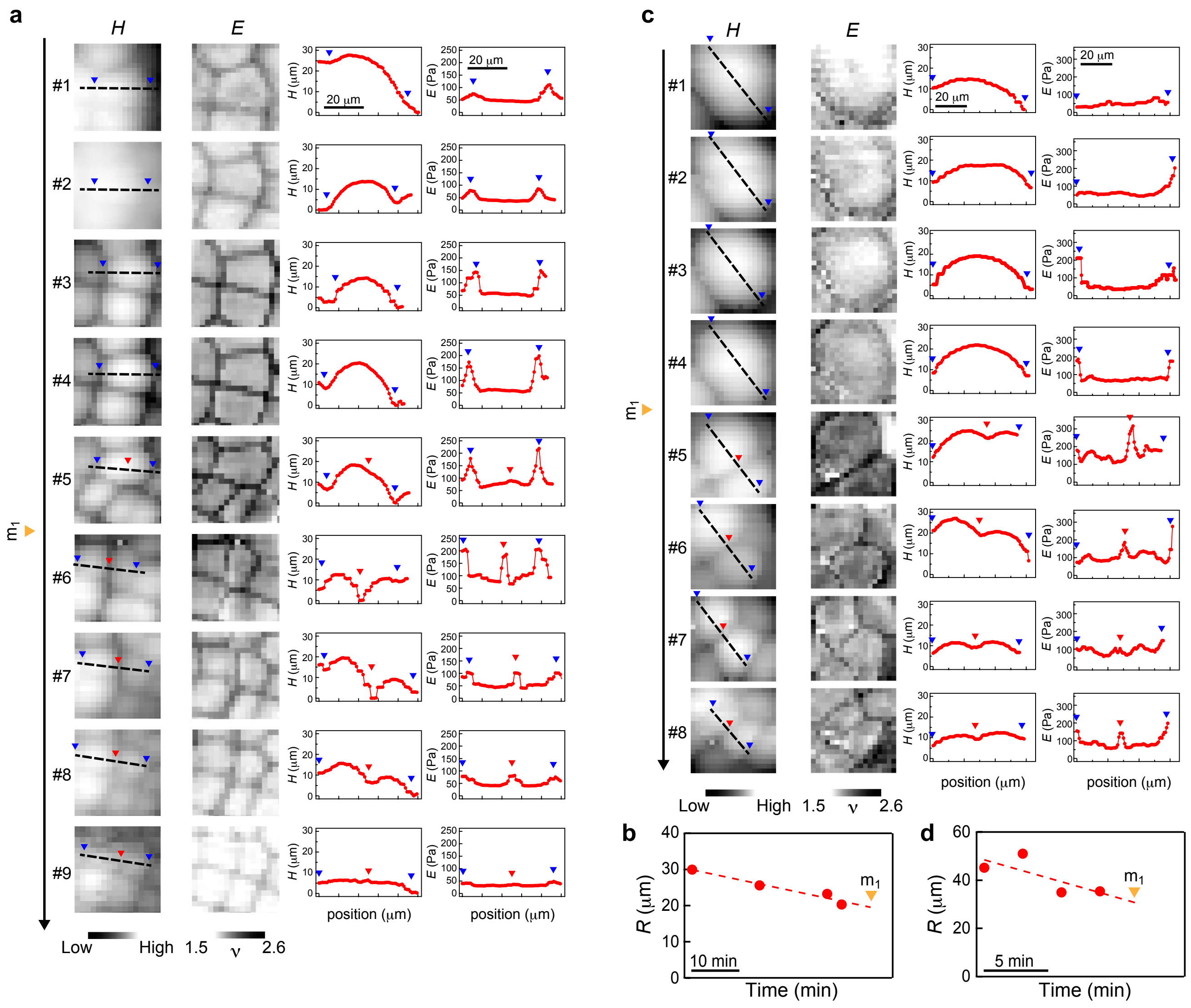

Supplementary Figure 2 Portions of *H* and *E* images of the embryo mapped in animal (**a**) and vegetal (**c**) hemispheres during cell division from the 76-cell to 112-cell stage and from the 32-cell to 44-cell stage, respectively, as depicted by the arrowheads (orange: m_1). The cleavage furrow depicted by the arrowhead (red) appeared from #5 in **a** and **c** where the arrowheads (blue) represent the cell-cell boundary. The cross-sectional cell apical shapes on major and minor axes, which were defined as *H* images in **a** and **c**, were used to estimate the curvature radius *R*. As a result, *R* of animal (**b**) and vegetal (**d**) cells from interphase to metaphase (#1-#4) in **a** and **c**, respectively, indicated cell rounding toward mitosis together with the increased in *E*.

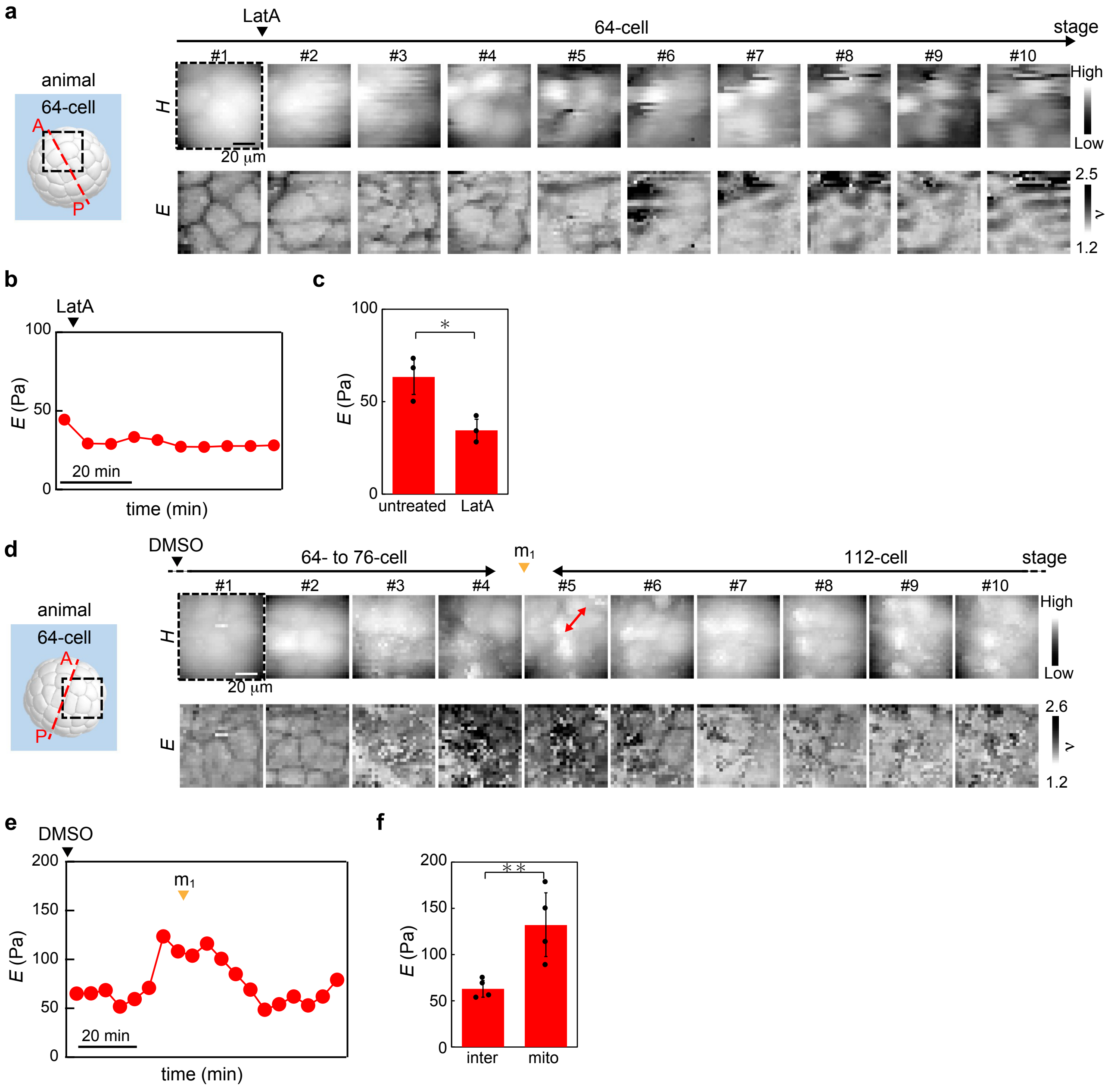

Supplementary Figure 3 Mapping the embryo in the animal hemisphere treated with LatA and DMSO. (**a**) *H* and *E* images of the embryo mapped in the animal hemisphere at the 64-cell stage treated with LatA as depicted by the arrowhead (black). Dotted square (black) in the 3D embryo model represents the measured region that approximately corresponded to image #1. (**b**) Plots of *E* around the centre of cells estimated from **a**. The addition of LatA is depicted by the arrowhead (black). (**c**) *E*-values of cells in untreated and LatA-treated conditions. Data are geometric mean ± s.d. from $n = 3$ embryos. *$p <$ 0.05. (**d**) Portions of *H* and *E* images of the embryo mapped in the animal hemisphere

from the 64-cell to 112-cell stage after addition of DMSO as depicted by the arrowhead (black). Dotted square (black) in the 3D embryo model represents the measured region that approximately corresponded to image #1. Double-headed arrow (red) represents the direction of cell division from the 76-cell to 112-cell stage, as depicted by the arrowhead (orange: m_1). (**e**) Plots of E around the centre of cells estimated from **d**. The addition of DMSO is depicted by the arrowhead (black). (**f**) E-values of cells at the timing of cell division (mito) and in interphase (inter) after adding DMSO. Data are geometric mean ± s.d. from $n = 4$ embryos. $^{**}p < 0.01$.

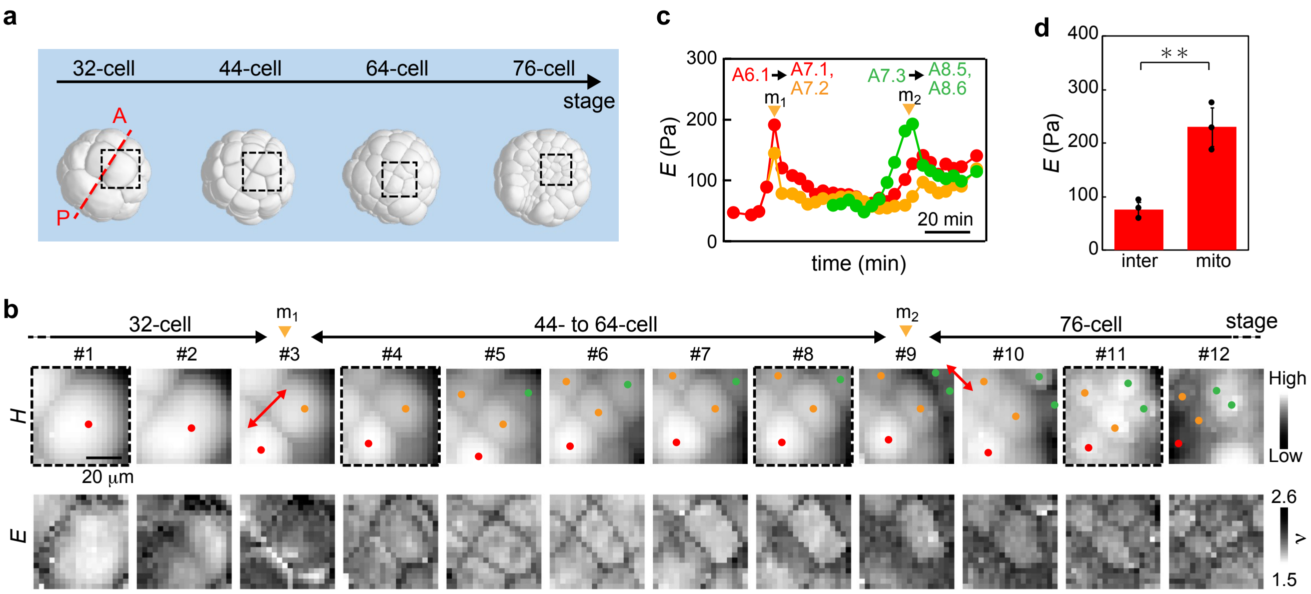

Supplementary Figure 4 Mapping the embryo in the vegetal hemisphere. (**a**) Morphological change in vegetal cells of the 3D model from the 32-cell to 76-cell stage. Dotted line (red) represents the anterior-posterior line of the embryo. Four dotted squares (black) represent the measured regions that approximately corresponded to the four images (#1, #4, #8, and #11) in **b**. (**b**) Portions of *H* and *E* images of the embryo mapped in the vegetal hemisphere from the 32-cell to 76-cell stage. Cell division occurred at times depicted by the arrowheads (orange: m_1 and m_2). Double-headed arrow (red) represents the direction of cell division. The colours of dots represent the following cells: A7.1 cell (red), A7.2 dell (orange), and A7.3, A8.5, and A8.6 cells (green). (**c**) Plots of *E* around the centre of cells estimated from **b** where the colours correspond to those in **b**. (**d**) *E*-values of A7.3 cell at the timing of cell division (mito) from the 64-cell to 76-cell stage and in the interphase in the 64-cell stage (inter). Data are geometric mean ± s.d. from $n = 3$ embryos. $^{**}p < 0.005$.

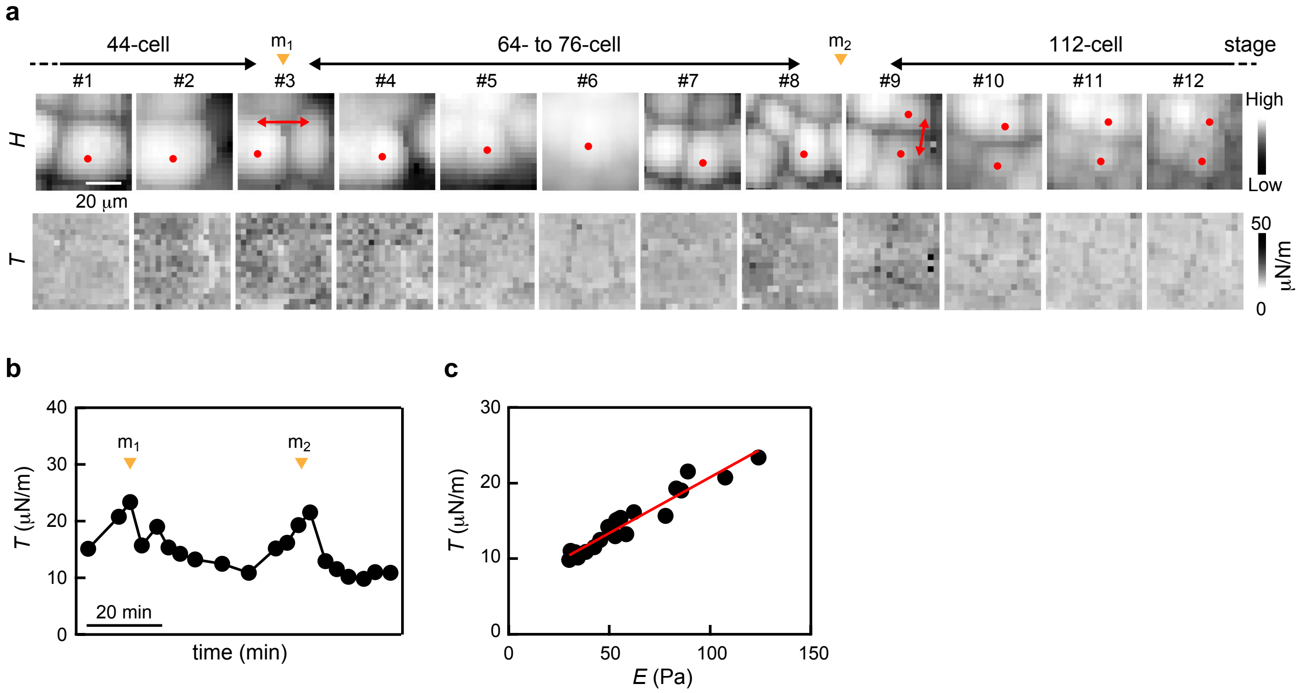

Supplementary Figure 5 Estimate of the apparent tension *T* in the embryo. (**a**) Portions *H* and *T* images of the embryo mapped in the animal hemisphere from the 44-cell to 112-cell stage, which is the same AFM data set in Fig. 3a. Two double-headed arrows represent the direction of cell division as depicted by the arrowheads (orange: m_1 and m_2). (**b**) Plots of *T* around the centre of cells as depicted by the dots (red) in **a**. (**c**) Plots of *E* versus and *T* showed a correlation coefficient of 0.97, which was estimated by a linear regression method in Excel.
