# Supplementary material for: Spatiotemporal dynamics of single cell stiffness in the early developing ascidian chordate embryo
Source: Commun Biol. 2021 Mar 16;4:341. doi: 10.1038/s42003-021-01869-w (PMC7966737; doi:10.1038/s42003-021-01869-w)
Supplement: Supplementary file 3 — Description of Additional Supplementary Files [file 42003_2021_1869_MOESM3_ESM.pdf]

Description of Additional Supplementary Files

File name: Supplementary Movie 1

Description: Live-imaging of F-actin-binding protein, lifeact-GFP (top) and membrane probe, FM4-64 (bottom) during two rounds of cell division from 44-cells to 112-cells.

File name: Supplementary Movie 2

Description: Confocal section images of dividing cells expressing lifeact-GFP (bottom) and cell membrane stained by FM4-64 (top) from 76-cells.

File name: Supplementary Data 1

Description: All source data underlying the graphs and charts presented in the main figures.
