# Supplementary material for: Spatiotemporal dynamics of single cell stiffness in the early developing ascidian chordate embryo
Source: Commun Biol. 2021 Mar 16;4:341. doi: 10.1038/s42003-021-01869-w (PMC7966737; doi:10.1038/s42003-021-01869-w)
Supplement: Supplementary file 7 — Reporting Summary [file 42003_2021_1869_MOESM7_ESM.pdf]

nature research

Corresponding author(s): Takaharu Okajima

Last updated by author(s): Jan 20, 2021

Reporting Summary

Nature Research wishes to improve the reproducibility of the work that we publish. This form provides structure for consistency and transparency in reporting. For further information on Nature Research policies, see our Editorial Policies and the Editorial Policy Checklist.

Statistics

For all statistical analyses, confirm that the following items are present in the figure legend, table legend, main text, or Methods section.

n/a	Confirmed	
☐	☒	The exact sample size (*n*) for each experimental group/condition, given as a discrete number and unit of measurement
☐	☒	A statement on whether measurements were taken from distinct samples or whether the same sample was measured repeatedly
☐	☒	The statistical test(s) used AND whether they are one- or two-sided<br>*Only common tests should be described solely by name; describe more complex techniques in the Methods section.*
☒	☐	A description of all covariates tested
☒	☐	A description of any assumptions or corrections, such as tests of normality and adjustment for multiple comparisons
☐	☒	A full description of the statistical parameters including central tendency (e.g. means) or other basic estimates (e.g. regression coefficient) AND variation (e.g. standard deviation) or associated estimates of uncertainty (e.g. confidence intervals)
☐	☒	For null hypothesis testing, the test statistic (e.g. F, t, r) with confidence intervals, effect sizes, degrees of freedom and P value noted<br>*Give P values as exact values whenever suitable.*
☒	☐	For Bayesian analysis, information on the choice of priors and Markov chain Monte Carlo settings
☒	☐	For hierarchical and complex designs, identification of the appropriate level for tests and full reporting of outcomes
☐	☒	Estimates of effect sizes (e.g. Cohen's *d*, Pearson's *r*), indicating how they were calculated

Our web collection on statistics for biologists contains articles on many of the points above.

Software and code

Policy information about availability of computer code

Data collection	Atomic force microscopy (AFM) data were acquired using program developed in LabVIEW (version 2013, National Instruments).
Data analysis	Igor Pro (version 8, WaveMetrics) and Image J (version 1.52, NIH) were used for the AFM data analysis. Microsoft Excel (version 16) was used for statistical analysis.

For manuscripts utilizing custom algorithms or software that are central to the research but not yet described in published literature, software must be made available to editors and reviewers. We strongly encourage code deposition in a community repository (e.g. GitHub). See the Nature Research guidelines for submitting code & software for further information.

Data

Policy information about availability of data

All manuscripts must include a data availability statement. This statement should provide the following information, where applicable:

- Accession codes, unique identifiers, or web links for publicly available datasets
- A list of figures that have associated raw data
- A description of any restrictions on data availability

All the data for generating the graphs in all the main figures are included in Supplementary Data. The image sets in the main figures are available from the corresponding author on reasonable request.

Field-specific reporting

Please select the one below that is the best fit for your research. If you are not sure, read the appropriate sections before making your selection.

☒ Life sciences ☐ Behavioural & social sciences ☐ Ecological, evolutionary & environmental sciences

For a reference copy of the document with all sections, see nature.com/documents/nr-reporting-summary-flat.pdf

Life sciences study design

All studies must disclose on these points even when the disclosure is negative.

Sample size	At least three independent time-lapse AFM measurements were performed in every condition.
Data exclusions	No data points were excluded from the AFM analysis.
Replication	The findings described in the paper were reproducible across multiple experiments.
Randomization	Not applicable
Blinding	Not applicable

Reporting for specific materials, systems and methods

We require information from authors about some types of materials, experimental systems and methods used in many studies. Here, indicate whether each material, system or method listed is relevant to your study. If you are not sure if a list item applies to your research, read the appropriate section before selecting a response.

Materials & experimental systems

n/a	Involved in the study	
☒	☐	Antibodies
☒	☐	Eukaryotic cell lines
☒	☐	Palaeontology and archaeology
☐	☒	Animals and other organisms
☒	☐	Human research participants
☒	☐	Clinical data
☒	☐	Dual use research of concern

Methods

n/a	Involved in the study	
☒	☐	ChIP-seq
☒	☐	Flow cytometry
☒	☐	MRI-based neuroimaging

Animals and other organisms

Policy information about studies involving animals; ARRIVE guidelines recommended for reporting animal research

Laboratory animals	Not applicable
Wild animals	Adult Ciona intestinalis type A (Ciona robusta) collected in the vicinity of the Maizuru Fisheries Research Station (Kyoto University) and Misaki Marine Biological Station (The University of Tokyo) through the National BioResource Project (NBRP, Japan) were shipped. Eggs and sperm were obtained by dissection of the ascidian gonoducts. Unfertilised eggs were dechorionated by natural seawater (Nihon Aquarium, Japan) containing 1.0% sodium thioglycolate (Wako Pure Chemical Industries Ltd., Japan) and 0.05% actinase E (Rikaken Pharmaceutical Co. Ltd., Japan) and then artificially inseminated.
Field-collected samples	The samples shipped were maintained in sea water at 18°C and used for experiments within three days.
Ethics oversight	No ethical approval was required for the samples.

Note that full information on the approval of the study protocol must also be provided in the manuscript.
