# Supplementary material for: Spatiotemporal dynamics of single cell stiffness in the early developing ascidian chordate embryo
Source: Commun Biol. 2021 Mar 16;4:341. doi: 10.1038/s42003-021-01869-w (PMC7966737; doi:10.1038/s42003-021-01869-w)
Supplement: Supplementary file 1 — Peer Review File [file 42003_2021_1869_MOESM1_ESM.pdf]

Reviewers' comments:

Reviewer #1 (Remarks to the Author):

This paper presents a study on the investigation of the stiffness of cells in ascidian embryos from the fertilised egg to the stage before gastrulation. The authors found that cell stiffness exhibited a remarkable increase at the onset of cell division and then decreased after cell division. They conclude that the mechanical properties of early embryonic cells are regulated specifically at the single cell level. They also performed a study on the ROCK inhibitor Y-27632, finding that the treatment did not completely suppress the increase in E at the timing of cell division.
Overall, the study is very interesting and new in the field. The experiments are well performed and clearly explained. However, I have some concerns regarding the description and presentation of some data; moreover some points need to be clarified.

- For the AFM experiments, the authors customized a rectangular cantilever arranging two silica beads from the apex of the AFM tip with epoxy glue. While the authors only show a schematic representation of the cantilever in Figure 1, there is a need of a clearer visualization of the tip. The authors should add a microscopic image of the customized cantilever (e.g. SEM) to show the reader the final geometry of the tip.
- In the paragraph "Cells stiffen at the timing of initial symmetrical cell division" at page 5, the authors claim that "the increase in E at the timing of cell division was associated with the formation of cortical actin filaments". Before the authors could claim this, there is a clear need of a quantitative fluorescent analysis of actin filaments.
The latrunculin test they performed only prove that actin filaments are the main responsible of the cell stiffness but does not explain the increase of E at the timing of cell division.
- The treatment with Rho kinase (ROCK) inhibitor Y-27632 deserves a clearer explanation.
The authors should describe the reasons why this specific inhibitor has been chosen and report more literature on this choice (also in the introduction).
Moreover, further explanations need to be given in the paragraph "Chemical treatments" in Materials and Methods. After how many minutes from the treatment AFM measurements were performed?

Minor issues:
Figure 1: in Figure 1b a scale bar is needed for the optical image and in the AFM Young's modulus image the values should be reported. Which values correspond to soft and hard, as reported in the figure?

Reviewer #2 (Remarks to the Author):

In the present study Fujii and colleagues used Atomic Force Microscopy (AFM) to measure cell stiffness (Young's modulus E) in ascidian embryos during early development, from fertilization to onset of gastrulation. The authors found that during initial cleavage divisions stages and subsequently in the animal hemisphere cells presented a characteristic increase in stiffness concomitant with cell rounding at the onset of division followed by a decrease as cells completed their divisions. This pattern was dependent on the integrity of a cortical actin meshwork but not on the activity of the ROCK pathway. Instead, in the vegetal hemisphere the authors report a different and more heterogenous pattern of cell stiffening with some cells not decreasing their stiffness after cell division and others increasing it without division. Stiffening in the vegetal hemisphere was dependent on the ROCK pathway and associated to the characteristic MyoII-dependent process of apical constriction occurring in this side of

the embryo.

In this study the authors report interesting new measurements of cell stiffness during early development of ascidian embryos. Such data have been lacking despite the many studies focusing on the mechanics of ascidian embryos morphogenesis. Thus, this study can potentially represent a significant contribution to the field. However, at present the paper suffers from few major shortcomings linked to over-interpretation of certain results and to sample size that need to be resolved to grant publication.
Listed below I provide my major criticisms, a list sentences I found confusing and a few suggestions to improve the manuscript..

Major concerns:

1. Role of cortical actin filaments in the increase of cell stiffness. Based on their data in Fig.2d-e and Suppl. Fig.3 the authors conclude that the formation and release of an actin cortex is responsible of the observed changes in E upon cell division, thus suggesting that a specific remodelling of the actin cortex drives cell stiffening. However, the estimated E is a measure of total stress which depends also on cortical tension and hydrostatic pressure, as the authors refer to in the discussion (pp.11). Therefore, the measured increase in E could also be interpreted as due an increase in hydrostatic pressure resisted by a constant non-remodelling actin cortex that would be put under tension. Disruption of the actin cortex by Lat-A treatment would also in this case reduce stiffness as cell swelling would no longer be resisted. In the absence of additional evidence showing a remodelling of the actin cortex concomitant with the increase in E, the authors cannot conclude that this is due to actin remodelling.

2. Different mechanical behaviours in the vegetal hemisphere. In the vegetal hemisphere the authors describe that some cells (B 6.1) maintain high stiffness after completing cell division while others increase stiffness without entering cell division (A7.1 and A7.2). However, in both cases stiffening was decreased by inhibiting ROCK and thus apical MyoII which begins to accumulate in these cells at the 64-cells stage and drives apical constriction. This indicates that in both cases the measured increase in stiffness is due to apical MyoII contractility which may occur immediately after cell divisions for some cells (e.g. in the case of cells B6.1) or in the middle of interphase for others (e.g. A7.1 and A7.2). It is thus unclear why the authors interpret this as two distinct mechanical behaviors (abstract and discussion pp.12).

3. Sample size. The sample size of the measurements is never indicated in the manuscript, figures and figure legends. How many cells and how many embryos were used to estimate E in the graphs in Figs. 2c, 2e, 3c, 3f, 4c, 4g, 5b, 5d and Suppl. Figs. 3b, 3d and 4c? Also, are Fig.3d and 4e mere summary schematics or are they based on real measurements? If so from how many cells and embryos? It would be good to provide a sense of the variability of the measurements within a population of similar cells across different embryos. For instance, for the comparison of cell stiffnesses in the ventral hemisphere (Fig.4e and text pp.9) it would be good to provide the measurements in the form of individual data points or box plots.

4. Possible data inconsistencies. I noticed some inconsistencies in the data across different panels where values are supposed to be similar.
- Fig.2c compared to Fig.5b: the authors claim that the treatment with Nocodazole had no impact on cell stiffening observed during the early cleavage divisions (pp.10), however while in Fig.2c E ranges from values between 50 and 300 Pa in Fig.5b E ranges from values from 50 to 200 Pa. Similar observation can be made for Fig.5d (E ranging from 20 to 80 Pa) compared to Fig.3c (E ranging from 50 to 100-120 Pa).

- Fig.4g compared to Fig.4c: In Fig.4g (before Y-27632 treatment) the baseline value of E before m1 is around 50 Pa and the peak value at m1 is around 200 Pa while in Fig.4c the baseline value of E before m1 is around 100 Pa and the peak value at m1 is around 400 Pa. Furthermore, the scaling of the E value in the images presented in the associated Fig.4b and Fig.4f is not the same. This makes the visual comparison impossible.
How do the authors explain such differences in conditions expected to yield similar values?
It would be preferable to compare these conditions showing individual cell or embryos data plots and/or box plots at mitosis and interphase.

5. Last result section on cell cycle arrest: The last section on cell cycle provides interesting additional data on the regulation of stiffening at cell division, but it appears misplaced in the flow of the manuscript. I find that these data would more appropriately be described after the results sections 2 and 3 describing cell stiffening at cell divisions during cleavage divisions and in the animal hemisphere.

Minor issues:

- The cells the authors refer to as A7.1, A7.2, A7.3, A7.5, B 7.1, A8.5, A8.6, B8.6 and B8.5 are indicated only in the summary schematics in Fig.4e but not in the initial schematics in Fig.4a which is the first the reader is confronted with. Furthermore, even in Fig.4e cells A6.1, B6.1 are not indicated and one has to deduce their position in the schematics. This makes the reading of the presented data quite difficult. It would be useful to indicate the cell IDs throughout in the schematics and images presented in Fig.4.

Complicated and/or unclear sentences:

- pp.4: "<i>Consequently, the vegetal cells displayed a cell-to-cell mechanical variation that was intrinsically different from an in vitro cell monolayer showing a long-range spatial correlation over adjacent cells through cell-cell mechanical interactions</i>"
What do the authors mean by a mechanical cell-to-cell variation that is "intrinsically" different from that in cell monolayers? Do they refer to sharper differences in the embryo as compared to cell monolayers in vitro? Also, can the authors exclude that the stiffening observed in the vegetal side of the embryo is not due to (at least in part) to collective effects due force integration across cells? Measuring E in isolated cells (i.e. after embryo dissociation or by lowering mechanical cell-cell coupling through adherens junction) would allow them to make this point.

- pp.8: "<i>Interestingly, the stiffening of A7.1 and A7.2 cells occurred in the subcellular region and not with accompanying cell division (#2 and #3 arrows in Fig. 4d), and the rate of cell stiffening was different between them (Fig. 4c and Supplementary Fig. 4c).</i>" It's not clear which subcellular region the authors refer to. In Fig.4d it is hard to localize the cells the authors refer to and visualize their cell outline. It would be useful to draw in the figure cell outlines and indicate with arrowheads the regions to look.

- pp.9 "<i>When entering the 76-cell stage, the A7.2 cell was softer than the surrounding cells such as A7.1, A8.5-6, and A8.13-14 cells (#4 in Fig. 4d), although these cells attained stiffness higher than in interphase of the 44-cell stage (Fig. 4c).</i>". The sentence appears incomplete. "<i>These cells attained stiffness higher than</i>" what?

pp.12-13: "<i>We noticed that no apparent cell-to-cell variation in myosin density in apical regions has been observed in previous studies by immunofluorescence, implying that the apical actomyosin

network is remodelled without changing the myosin density and facilitates a mechanically stabilised structure.</i>" It is unclear to me what the authors want to communicate/suggest with this sentence. Are they trying to contrast an uniform MyoII density across the field of apically constricting cells with the difference in E that they measure? If so, what are they trying to suggest?

Responses to Reviewers' Comments

Manuscript title: Spatiotemporal dynamics of single cell stiffness in the early developing chordate embryo
Manuscript ID: COMMSBIO-20-1493-T
Author(s): Yuki Fujii, Wataru C. Koizumi, Taichi Imai, Megumi Yokobori, Tomohiro Matsuo, Kotaro Oka, Kohji Hotta, and Takaharu Okajima

We thank the reviewers for their careful review and helpful comments. We have revised the manuscript in consideration of the reviewers' comments. The reviewers' comments are italicized, and our point-by-point responses are in regular font. Page and line numbers in brackets refer to those in the revised manuscript.

Reviewer #1:
- For the AFM experiments, the authors customized a rectangular cantilever arranging two silica beads from the apex of the AFM tip with epoxy glue. While the authors only show a schematic representation of the cantilever in Figure 1, there is a need of a clearer visualization of the tip. The authors should add a microscopic image of the customized cantilever (e.g. SEM) to show the reader the final geometry of the tip.

- Thank you very much for your comments and suggestions. We have added an optical microscopic image of the customized cantilever arranging two silica beads from the AFM tip with epoxy glue in the inset of Fig. 1a in the revised manuscript (**Figure 1 and Page 16, line 20– Page 17, line 1**).

- In the paragraph "Cells stiffen at the timing of initial symmetrical cell division" at page 5, the authors claim that "the increase in E at the timing of cell division was associated with the formation of cortical actin filaments". Before the authors could claim this, there is a clear need of a quantitative fluorescent analysis of actin filaments. The latrunculin test they performed only prove that actin filaments are the main responsible of the cell stiffness but does not explain the increase of E at the timing of cell division.

- We agree that the latrunculin test does not demonstrate that the actin filaments play an important role in the increase of *E* at the timing of cell division. Because no direct observation of cortical actin filaments in the early developing chordate embryo has been reported, we attempted to visualize actin filaments in a developing embryo by scanning laser confocal microscopy and observed clear recruitment and release of actin filaments in cortical regions of the animal hemisphere at the timing of cell division. Because live imaging of actin filaments in the initial symmetrical cell division could not be achieved due to a limitation of the experimental protocol, we have suggested that the increase in *E* at the timing of cell division was associated with the formation of cortical actin filaments in the animal hemisphere section (**page 8, lines 7–13, and Supplementary Figure 6**).

- *The treatment with Rho kinase (ROCK) inhibitor Y-27632 deserves a clearer explanation. The authors should describe the reasons why this specific inhibitor has been chosen and report more literature on this choice (also in the introduction).*

- Immunofluorescence revealed that the apical shrinkage in the vegetal hemisphere was relaxed and the apical and circum-apical accumulation of myosin was reduced as the embryo was treated with Rho kinase (ROCK) inhibitor Y-27632. This observation indicated that a Rho pathway controlled the accumulation of myosin in the apical region of the vegetal hemisphere before gastrulation. Thus, we investigated how the mechanical properties of embryonic cells in the vegetal hemisphere were affected by Y-27632 treatment. We have added this text to the Introduction section. (**page 4, lines 10–14**)

Moreover, further explanations need to be given in the paragraph "Chemical treatments" in Materials and Methods. After how many minutes from the treatment AFM measurements were performed?

- The time when chemicals were added to samples is indicated by arrowheads in Figures 2–5 and Supplementary Figure 3, which is also explained in the figure captions. To quantify the chemical dependence of *E*, we analysed the treated cells after incubation for at least 10 min with latA and 20 min with Y-27632 (**page 19, lines 3–5**).

Minor issues:
Figure 1: in Figure 1b a scale bar is needed for the optical image and in the AFM Young's modulus image the values should be reported. Which values correspond to soft and hard, as reported in the figure?

- We have added scale bars in the optical and AFM Young's modulus images of Figure 1b in the revised manuscript. In the figure legend, we have also explained that the values of soft and hard presented in Figure 1b are $\nu = 1.4$ and 2.8 of $\nu = \log_{10}E$ (Pa), respectively (**Figure 1**).

Reviewer #2:
Major concerns:
1. Role of cortical actin filaments in the increase of cell stiffness. Based on their data in Fig.2d-e and Suppl. Fig.3 the authors conclude that the formation and release of an actin cortex is responsible of the observed changes in E upon cell division, thus suggesting that a specific remodelling of the actin cortex drives cell stiffening. However, the estimated E is a measure of total stress which depends also on cortical tension and hydrostatic pressure, as the authors refer to in the discussion (pp.11). Therefore, the measured increase in E could also be interpreted as due an increase in hydrostatic pressure resisted by a constant non-remodelling actin cortex that would be put under tension. Disruption of the actin cortex by Lat-A treatment would also in this case reduce stiffness as cell swelling would no longer be resisted. In the absence of additional

evidence showing a remodelling of the actin cortex concomitant with the increase in E, the authors cannot conclude that this is due to actin remodelling.

- Thank you very much for your comments and suggestions. We agree that additional evidence is needed to conclude that the formation and release of an actin cortex are responsible for the observed changes in *E* upon cell division. Therefore, we attempted to visualize actin filaments in a developing embryo by scanning laser confocal microscopy and observed clear recruitment and release of actin filaments in cortical regions of the animal hemisphere at the timing of cell division. Taken together with our AFM observations, we have concluded that the increase in *E* at the timing of cell division was strongly associated with the formation of cortical actin filaments. In accordance with previous studies, the tension of cortical actin filaments balances the hydrodynamic pressure during mitosis. Therefore, our conclusion is not contradicted by the indication that the estimated *E* is a measure of total stress that also depends on cortical tension and hydrostatic pressure (**page 8, lines 7–13, and Supplementary Figure 6**).

2. Different mechanical behaviours in the vegetal hemisphere. In the vegetal hemisphere the authors describe that some cells (B 6.1) maintain high stiffness after completing cell division while others increase stiffness without entering cell division (A7.1 and A7.2). However, in both cases stiffening was decreased by inhibiting ROCK and thus apical MyoII which begins to accumulate in these cells at the 64-cells stage and drives apical constriction. This indicates that in both cases the measured increase in stiffness is due to apical MyoII contractility which may occur immediately after cell divisions for some cells (e.g. in the case of cells B6.1) or in the middle of interphase for others (e.g. A7.1 and A7.2). It is thus unclear why the authors interpret this as two distinct mechanical behaviors (abstract and discussion pp.12).

- We classified temporal changes in the stiffness, that is, mechanical behaviours measured by AFM, into three types, and two mechanical behaviours observed only in the vegetal hemisphere were due to the apical contraction of actomyosin as explained on page 12, lines 4–22 in the Discussion section of the previous manuscript. Thus, our explanations in the previous manuscript are the same as those commented on by the reviewer. However, we noticed that the term “found” in the abstract was misleading, and thus we have changed the term “found” to “observed” (**page 2, line 9**).

3. Sample size. The sample size of the measurements is never indicated in the manuscript, figures and figure legends. How many cells and how many embryos were used to estimate E in the graphs in Figs. 2c, 2e, 3c, 3f, 4c, 4g, 5b, 5d and Suppl. Figs. 3b, 3d and 4c? Also, are Fig.3d and 4e mere summary schematics or are they based on real measurements? If so from how many cells and embryos? It would be good to provide a sense of the variability of the measurements within a population of similar cells across different embryos. For instance, for the comparison of cell stiffnesses in the ventral hemisphere (Fig.4e and text pp.9) it would be good to provide the measurements in the form of individual data points or box plots.

- Figs. 2c, 2e, 5b, 5d, and Supplemental Fig. 3b and 3d in the previous manuscript showed plots of geometrically averaged *E* around the cell centre, which was estimated from all images analysed in Figs. 2b, 2d, 5b, 5d, and Supplemental Fig. 3a and 3c in the previous manuscript, respectively. Additionally, Figs. 3c, 3f, 4c, 4g, and Supplemental Fig. 4c in the previous manuscript showed plots of geometrically averaged *E* around the centre of cells indicated by the dots, which was estimated from all images analysed in Figs. 3b, 3e, 4b, 4f, and Supplemental Fig. 4b in the previous manuscript, respectively. This information was explained in the figure captions of the previous manuscript. In the revised manuscript, we have shown the quantifications of *E* (geometric mean ± s.d.) in Figs. 2d, 2g, 3d, 3h, 4c, 4f, 5d, 5e, 5f, 5k, and Supplemental Figs. 3c, 3f, and 4d together with the sample size (the number of embryos) and *p*-value estimated by the Student's t-test. We have also clearly explained the time evolution observed in the animal and vegetal hemispheres as summarized schematically in Figs. 3e and 5h, respectively (**Figures 2–5; Supplementary Figures 3 and 4; page 7, lines 13–17; page 8, lines 14–18; page 9, lines 2–12; page 9, line 15–page 11, line 16; Page 19, lines 3–5; page 20, lines 14–15**).

4. Possible data inconsistencies. I noticed some inconsistencies in the data across different panels where values are supposed to be similar.

- Fig.2c compared to Fig.5b: the authors claim that the treatment with Nocodazole had no impact on cell stiffening observed during the early cleavage divisions (pp.10), however while in Fig.2c E ranges from values between 50 and 300 Pa in Fig.5b E ranges from values from 50 to 200 Pa. Similar observation can be made for Fig.5d (E ranging from 20 to 80 Pa) compared to Fig.3c (E ranging from 50 to 100-120 Pa).

- Fig.4g compared to Fig.4c: In Fig.4g (before Y-27632 treatment) the baseline value of E before m1 is around 50 Pa and the peak value at m1 is around 200 Pa while in Fig.4c the baseline value of E before m1 is around 100 Pa and the peak value at m1 is around 400 Pa. Furthermore, the scaling of the E value in the images presented in the associated Fig.4b and Fig.4f is not the same. This makes the visual comparison impossible.

How do the authors explain such differences in conditions expected to yield similar values?

It would be preferable to compare these conditions showing individual cell or embryos data plots and/or box plots at mitosis and interphase.

- In the previous manuscript, we did not mention an important result showing that the magnitude of *E* values was largely varied among native embryo samples even under the same condition, which indicated embryo-to-embryo mechanical variation in a chordate sample. The variation was also likely sensitive to the temperature of seawater from which the native chordates samples were collected and the culture day. However, because the origin of the magnitude variation of *E* has not been elucidated, we estimated the average *E* of embryonic cells and the significant difference of *E* between different states or conditions in the present study (**page 5, line 18–page 6, line 2**).

5. Last result section on cell cycle arrest: The last section on cell cycle provides interesting additional data on the regulation of stiffening at cell division, but it appears misplaced in the flow of the manuscript. I find that these data would more appropriately be described after the results sections 2 and 3 describing cell stiffening at cell divisions during cleavage divisions and in the animal hemisphere.

- In accordance with the reviewer's comments, the section on cell cycle arrest has been placed just after the section describing cell stiffening at cell divisions during cleavage divisions and in the animal hemisphere (**page 8, line 20–page 9, line 12**).

Minor issues:

- *The cells the authors refer to as A7.1, A7.2, A7.3, A7.5, B 7.1, A8.5, A8.6, B8.6 and B8.5 are indicated only in the summary schematics in Fig.4e but not in the initial schematics in Fig.4a which is the first the reader is confronted with. Furthermore, even in Fig.4e cells A6.1, B6.1 are not indicated and one has to deduce their position in the schematics. This makes the reading of the presented data quite difficult. It would be useful to indicate the cell IDs throughout in the schematics and images presented in Fig.4.*

- In accordance with the reviewer's comments, we have added the cell IDs in Fig. 5a, g, and h of the revised manuscript (**Figure 5**).

Complicated and/or unclear sentences:

- *pp.4: "Consequently, the vegetal cells displayed a cell-to-cell mechanical variation that was intrinsically different from an in vitro cell monolayer showing a long-range spatial correlation over adjacent cells through cell-cell mechanical interactions"*

What do the authors mean by a mechanical cell-to-cell variation that is "intrinsically" different from that in cell monolayers? Do they refer to sharper differences in the embryo as compared to cell monolayers in vitro? Also, can the authors exclude that the stiffening observed in the vegetal side of the embryo is not due to (at least in part) to collective effects due force integration across cells? Measuring E in isolated cells (i.e. after embryo dissociation or by lowering mechanical cell-cell coupling through adherens junction) would allow them to make this point.

- What we wanted to explain in the sentence was that the cell-to-cell mechanical variation observed in the vegetal hemisphere has not been observed in an in vitro cell monolayer, which indicated that the mechanical properties of early embryonic cells were specifically regulated at the single cell level. However, we realized that the sentence was unsuitable in the Introduction section. Therefore, we have removed the sentence. Alternatively, we have explained our main findings in the Introduction section (**page 4, lines 15–22**).

- *pp.8: "Interestingly, the stiffening of A7.1 and A7.2 cells occurred in the subcellular region and not with accompanying cell division (#2 and #3 arrows in Fig. 4d), and the rate of cell stiffening was different between them (Fig. 4c and Supplementary Fig. 4c)." It's not clear which subcellular region the authors refer to. In Fig.4d it is hard to localize the cells the authors refer to and*

visualize their cell outline. It would be useful to draw in the figure cell outlines and indicate with arrowheads the regions to look.

- In accordance with the reviewer's comment, the subcellar region imaged by AFM has been depicted as a schematic of an embryo with the corresponding arrowhead and dots in Fig. 5g of the revised manuscript (**Figure 5**).

- *pp.9 "When entering the 76-cell stage, the A7.2 cell was softer than the surrounding cells such as A7.1, A8.5-6, and A8.13-14 cells (#4 in Fig. 4d), although these cells attained stiffness higher than in interphase of the 44-cell stage (Fig. 4c).*". The sentence appears incomplete. "*These cells attained stiffness higher than*" what?

- We have revised the manuscript to indicate that these cells attained stiffness higher than ~100 Pa in interphase of the 44-cell stage (**page 10, lines 18–19**).

pp.12-13: "We noticed that no apparent cell-to-cell variation in myosin density in apical regions has been observed in previous studies by immunofluorescence, implying that the apical actomyosin network is remodelled without changing the myosin density and facilitates a mechanically stabilised structure." It is unclear to me what the authors want to communicate/suggest with this sentence. Are they trying to contrast an uniform MyoII density across the field of apically constricting cells with the difference in E that they measure? If so, what are they trying to suggest?

- What we wanted to suggest in the sentence was that AFM provides a complementary approach to quantitative investigations of dynamic structural changes in embryonic cells during developmental processes. Taken together with the previous immunofluorescence and our AFM observations, we suggested that the apical actomyosin network is remodelled without changing the myosin density and facilitates a mechanically stabilized structure (**page 14, lines 3–8**).

Additionally, we made minor changes to the revised manuscript, such as correcting mistyped sentences and errors.

REVIEWERS' COMMENTS:

Reviewer #2 (Remarks to the Author):

In this revised manuscript the authors present new data and analysis (visualization of actin cortex dynamics during cell division) that substantially strengthen and support their claims. The explanations provided with the rebuttal letter and the new information integrated in the manuscript and figures address most of my criticisms and I can now recommend publication.
Nevertheless, I have few remaining suggestions that in my opinion would still improve the manuscript.

1) The authors insist on focusing on a classification of events based on temporal changes of stiffening and, consequently, on distinguishing 3 types of changes in E. While I understand this classification, I think that it is not useful and I find it even confusing. Instead I would rather focus on a classification of the events based on the mechanism (or dependency on the activity of the ROCK pathway), which, I think, is more useful and straight forward. I would, thus, only distinguish two types of events, stiffening due to cell divisions (dependent on an unknown pathway) and a stiffening due to apical MyoII. Then cells in the animal hemisphere are only use one mechanism while cells in the vegetal hemisphere use both.

2) The new data presented in Supplementary Figure 6 are very nice and represent a key addition to the manuscript. I would rather have this as main figure clearly showing the implication of the actin cortex in stiffening instead of the data in main figure 6. Furthermore, it would be useful to include the movies of the data presented in Supplementary figure 6b and Supplementary figure 6d.

3) The sentence listed below is purely speculative and need to be toned down:
- pp7, lines 5-7: "The lower value of E measured in embryonic cells implies that cortical actin filaments formed in embryonic cells during the initial developing process are more mechanically flexible and fragile rather than in cells in vitro." In the manuscript there are no direct measurement of actin filament/actin network stiffness. The lower value of E measured in embryo might also depend on other factors such hydrostatic pressure or cortical tension of cells.

Responses to Reviewers’ Comments

Manuscript title: Spatiotemporal dynamics of single cell stiffness in the early developing chordate embryo
Manuscript ID: COMMSBIO-20-1493A
Author(s): Yuki Fujii, Wataru C. Koizumi, Taichi Imai, Megumi Yokobori, Tomohiro Matsuo, Kotaro Oka, Kohji Hotta, and Takaharu Okajima

We thank the reviewer for their careful review and helpful comments. We have revised the manuscript in consideration of the reviewer’s comments. The reviewer’s comments are italicized, and our point-by-point responses are in regular font. Page and line numbers in brackets refer to those in the revised manuscript.

Reviewer #2:
1) The authors insist on focusing on a classification of events based on temporal changes of stiffening and, consequently, on distinguishing 3 types of changes in E. While I understand this classification, I think that it is not useful and I find it even confusing. Instead I would rather focus on a classification of the events based on the mechanism (or dependency on the activity of the ROCK pathway), which, I think, is more useful and straight forward. I would, thus, only distinguish two types of events, stiffening due to cell divisions (dependent on an unknown pathway) and a stiffening due to apical MyoII. Then cells in the animal hemisphere are only use one mechanism while cells in the vegetal hemisphere use both.

- Thank you very much for your comments and suggestions. In accordance with the reviewer’s comments, we revised the manuscript so that the temporal changes in cell stiffening are classified into two types which are associated with the Rho kinase (ROCK) pathway and other unknown pathway (page 2, lines 1–14 and page 13, lines 1–12 in the revised manuscript).

2) The new data presented in Supplementary Figure 6 are very nice and represent a key addition to the manuscript. I would rather have this as main figure clearly showing the implication of the actin cortex in stiffening instead of the data in main figure 6. Furthermore, it would be useful to include the movies of the data presented in Supplementary figure 6b and Supplementary figure 6d.

- In accordance with the reviewer’s comments, Supplementary Figure 6 has been placed in Figure 4 as main figure. We have also showed the movies of the data presented in Supplementary Figure 6b and d of the previous manuscript as Supplementary Movies in the revised manuscript.

3) The sentence listed below is purely speculative and need to be toned down: - pp7, lines 5-7: "The lower value of E measured in embryonic cells implies that cortical actin

filaments formed in embryonic cells during the initial developing process are more mechanically flexible and fragile rather than in cells in vitro." In the manuscript there are no direct measurement of actin filament/actin network stiffness. The lower value of E measured in embryo might also depend on other factors such hydrostatic pressure or cortical tension of cells.

- We agree that the sentence in page 7, lines 5-7 of the previous manuscript is speculative and
  there is no direct measurement of actin filament/actin network stiffness. Therefore, we have removed this sentence in the revised manuscript.

Additionally, we have included an additional experimental data point in Fig. 6k and Supplementary Fig. 4d in the revised manuscript to show the statistics in the accordance with the Final Revision Instructions. We have also made minor changes to the revised manuscript, such as correcting mistyped sentences and errors.
